# Leaf-, panel- and latex-expressed sequenced tags from the rubber tree (*Hevea brasiliensis*) under cold-stressed and suboptimal growing conditions: the development of gene-targeted functional markers for stress response

**DOI:** 10.1007/s11032-014-0095-2

**Published:** 2014-04-29

**Authors:** Carla C. Silva, Camila C. Mantello, Tatiana Campos, Livia M. Souza, Paulo S. Gonçalves, Anete P. Souza

**Affiliations:** 1 Centro de Biologia Molecular e Engenharia Genética (CBMEG), Universidade Estadual de Campinas (UNICAMP), Cidade Universitária Zeferino Vaz, CP 6010, Campinas, SP CEP 13083-970 Brazil; 2 Centro de Pesquisa Agroflorestal do Acre (CPAFAC), Embrapa, Rodovia BR-364, km 14, CP 321, Rio Branco, AC CEP 69900-970 Brazil; 3 Instituto Agronômico de Campinas (IAC), CP 28, Campinas, SP CEP 13012-970 Brazil; 4 Departamento de Biologia Vegetal, Instituto de Biologia, Universidade Estadual de Campinas (UNICAMP), Cidade Universitária Zeferino Vaz, CP 6109, Campinas, SP CEP 13083-970 Brazil

**Keywords:** *Hevea*, Rubber tree, cDNA library, Molecular markers, Microsatellite, SNPs

## Abstract

*Hevea brasiliensis* is a native species of the Amazon Basin of South America and the primary source of natural rubber worldwide. Due to the occurrence of South American Leaf Blight disease in this area, rubber plantations have been extended to suboptimal regions. Rubber tree breeding is time-consuming and expensive, but molecular markers can serve as a tool for early evaluation, thus reducing time and costs. In this work, we constructed six different cDNA libraries with the aim of developing gene-targeted molecular markers for the rubber tree. A total of 8,263 reads were assembled, generating 5,025 unigenes that were analyzed; 912 expressed sequence tags (ESTs) represented new transcripts, and two sequences were highly up-regulated by cold stress. These unigenes were scanned for microsatellite (SSR) regions and single nucleotide polymorphisms (SNPs). In total, 169 novel EST-SSR markers were developed; 138 loci were polymorphic in the rubber tree, and 98 % presented transferability to six other *Hevea* species. Locus duplication was observed in *H. brasiliensis* and other species. Additionally, 43 SNP markers in 13 sequences that showed similarity to proteins involved in stress response, latex biosynthesis and developmental processes were characterized. cDNA libraries are a rich source of SSR and SNP markers and enable the identification of new transcripts. The new markers developed here will be a valuable resource for linkage mapping, QTL identification and other studies in the rubber tree and can also be used to evaluate the genetic variability of other *Hevea* species, which are valuable assets in rubber tree breeding.

**Electronic supplementary material:**

The online version of this article (doi:10.1007/s11032-014-0095-2) contains supplementary material, which is available to authorized users.

## Introduction

Rubber tree [*Hevea brasiliensis* (Willd. ex Adr. de Juss.) Muell-Arg.], a native species of the Amazon rainforests, is the primary commercial source of natural rubber. *H. brasiliensis* is a diploid (2*n* = 36, *n* = 18), perennial, cross-pollinated and monoecious tropical tree that belongs to the Euphorbiaceae family. The genus *Hevea* is composed of 11 inter-crossable species, of which *H. brasiliensis* is the most economically important (Pires et al. 2002; Gonçalves et al. 1990). Natural rubber is used in a large variety of products due to its flexibility, resistance, plasticity, impermeability and insulating properties (Mooibroek and Cornish 2000). In 2012, 11.327 million tons of natural rubber were produced worldwide to meet a demand of 11.005 million tons (International Rubber Study Group (IRSG) 2013); by the year 2020, economists predict that the demand for natural rubber will surpass its production by thousands of tons (Burger and Smith 1997).

The Amazon Basin presents a suitable climate for crop development, but the occurrence of South American Leaf Blight (SALB), which is caused by the fungus *Microcyclus ulei* (P. Henn) v. Arx, limits rubber production in the area (Pushparajah 2001). This problem has led to the expansion of rubber plantations to suboptimal areas, such as northeastern India, Vietnam, southern China and the southern plateau of Brazil. In addition to new conditions for crop development, these new areas of production often present stressful conditions, such as low temperatures, high altitudes, typhoons and dry periods, and all of these factors affect latex production (Pushparajah 1983; Priyadarshan and Gonçalves 2003). Rubber breeding programs aim to identify clones that are adapted to these stress conditions (Priyadarshan and Gonçalves 2003). However, rubber tree breeding is time-consuming and expensive because it can take more than 20 years to develop a new variety (Gonçalves and Fontes 2012). The generation of molecular markers can enable the early detection of the target genotype by marker-assisted selection (MAS), thus reducing the length of the breeding period and its costs.

Expressed sequence tags (ESTs) are a powerful tool for genetic studies such as gene identification, tissue expression profiling and gene mapping; ESTs are also a rich source of molecular markers (Varshney et al. 2005a; Harbers 2008). Microsatellite regions (SSRs; Varshney et al. 2005a) and single nucleotide polymorphisms (SNPs; Rafalski 2002) are the most important and most widely used markers that can be developed from ESTs. Unlike genomic microsatellites, EST-SSRs are likely to be embedded in functional gene sequences because they are gene-targeted markers that have the potential to become functional markers (Andersen and Lübberstedt 2003; Varshney et al. 2005a). Although EST-derived SSR markers are less polymorphic than genomic SSRs, they are better defined (Varshney et al. 2005a) and exhibit higher rates of transferability to related species (Varshney et al. 2005b; Feng et al. 2009). SNPs are highly abundant in the genome and can be used for the construction of high-resolution maps due to their frequency and distribution (Andersen and Lübberstedt 2003; Gaur et al. 2012). EST-derived SNP markers are also gene-targeted and can be directly involved in the expression of a desirable trait; therefore, they are a tool for marker-assisted selection (Liu et al. 2012; Xia et al. 2012) and are useful for genetic studies such as candidate gene mapping and gene-based association studies.

*H. brasiliensis* EST studies have been carried out over the last 10 years (Ko et al. 2003; Chow et al. 2007), but large-scale EST studies of the rubber tree have only been initiated in the last few years (Xia et al. 2011; Triwitayakorn et al. 2011; Li et al. 2012). The development of EST-derived SSR markers for the rubber tree has also only recently been reported (Feng et al. 2009; Triwitayakorn et al. 2011; Li et al. 2012), and only ten SNP markers have been reported thus far (Pootakham et al. 2011). In the present work, cDNA libraries of cold-stressed clones and different tissues from the rubber tree were constructed for the development of EST-SSR and SNP markers.

## Methods

### Plant materials

Clonal graftings of *Hevea brasiliensis* clones PB 217, PR 255, GT 1 and IAN 873 were subjected to a 24-h cold treatment in a Thermo Forma Diurnal Growth Chamber (model 3740; Thermo Scientific Inc., USA) and maintained at 8 °C with a 12-h photoperiod. This treatment was performed to promote the expression of genes involved in cold response and for the development of molecular markers related to this stress condition. Clones PB 217 (high rubber yield potential and cold sensitive) and PR 255 (tolerant to injury and cold) are the parents of a mapping population (Souza et al. 2013), and clones GT 1 and IAN 873 showed cold tolerance in the field (Gonçalves PS, personal communication). The leaves were wrapped in tinfoil prior to collection to prevent transcript redundancy. The leaves were sampled at intervals of 0, 6, 10 and 24 h; immediately frozen in liquid nitrogen; and stored at −80 °C until use. For the panel and latex libraries, samples were collected from 16-year-old tree clones of PB 217, PR 255, GT 1, PB 235, RRIM 701 and IAN 873, and leaves of the same clones were collected from the rubber tree germplasm. Clones GT 1, PB 235 and RRIM 701 are the parents of two mapping populations that are being evaluated in our laboratory, and all clones used are recommended for cultivation in São Paulo State (Gonçalves PS, personal communication). This number of clones was used to increase the chance of detecting SNPs related to stress conditions for mapping in the F1 populations under evaluation. All of the samples were frozen immediately on dry ice and stored at −80 °C prior to RNA extraction.

To characterize the microsatellite markers, 18 contrasting *H. brasiliensis* genotypes were selected, including clones PB 217, PR 255, GT 1, PB 235 and RRIM 701. Furthermore, six species from the genus *Hevea* were included to assay the transferability of the SSR markers. The SNP markers were characterized using the clones listed above in addition to 18 other *H. brasiliensis* genotypes. Thus, a total of 36 clones were used to validate the polymorphic positions (Online Resource 1—Table S1). All of the *H. brasiliensis* samples were collected at the Rubber Research Center of the Agronomic Institute of Campinas (IAC), Votuporanga, São Paulo, Brazil (latitude: 20°25′22″S; longitude: 49°58′22″W), which is a suboptimal region for rubber plantations. The samples of the other species were obtained from the West Amazon Agroforestry Research Center (Embrapa Amazônia Ocidental), Amazonas, Brazil.

### RNA preparation, cDNA library construction and EST generation

Total RNA was extracted from the leaves, panel and latex following the protocol described by Chang et al. (1993) and treated with RNAse-free DNAse I (Qiagen Inc., USA). Equal amounts of total RNA were pooled (up to 5 μg) according to tissue (leaf, panel and latex) and time of sampling (6-, 10- and 24-h cold treatment). The In-fusion SMARTer cDNA Library Construction kit (Clontech Laboratories Inc., USA) was used to construct the cDNA libraries according to the manufacturer’s instructions. The ligation mixtures were transformed into electrocompetent *Escherichia coli* DH10B cells, and colonies were selected using LB-ampicillin plates containing IPTG (isopropylthio-β-galactoside) and X-gal (5-bromo-4-chloro-3-indolyl-β-d-galactoside). The insert fragment sizes of 15 positive clones from each library were analyzed by PCR amplification using M13 primers.

Sequencing (10-μL reaction mixtures) was carried out from the 5′ end of the inserts using M13 primers and the Big Dye Terminator 3.1 Cycle Sequencing kit (Applied Biosystems Inc., USA). The sequencing reactions were analyzed in a 3500XL DNA ABI PRISM Automatic Sequencer (Applied Biosystems Inc., USA).

### DNA extraction

Genomic DNA was extracted from the lyophilized leaf tissues using a modified CTAB method (Doyle and Doyle 1987). The quality and concentration of the DNA were assessed by 1 % agarose gel electrophoresis.

### EST sequence processing and analysis

PHRED (Ewing and Green 1998) was used to perform vector and poly(A) removal and to trim low-quality segments. CLC Genomics Workbench 4 (CLC bio A/S, Denmark) and ChromasPro 1.5 (Technelysium Pty Ltd., Australia) software were used to assemble the high-quality EST sequences into contigs and singletons. A similarity comparison was performed with the ESTs (contigs and singletons) using the BLAST2GO program (Conesa et al. 2005) to search the National Center for Biotechnology Information (NCBI) non-redundant (nr) protein database. Additionally, the BLAST2GO program was used with default parameters to obtain the Gene Ontology (GO; Ashburner et al. 2000) terms for the molecular function, biological process and cellular component categories and to identify the metabolic pathways using the Kyoto Encyclopedia of Genes and Genomes (KEGG) database (Ogata et al. 1999). Open reading frames (ORFs) were predicted using the OrfPredictor program (Min et al. 2005). All processed EST sequences were deposited into the NCBI dbEST database under accession numbers JZ536145 to JZ544407.

### Quantitative RT-PCR analysis

Expression analysis of the cold-stressed cDNA libraries most represented unigenes was performed by quantitative RT-PCR. For the analysis of individual samples, 1 μg of total RNA was used for cDNA synthesis. For the combined samples analysis, equal amounts of total RNA were pooled according to time of sampling (0-, 6-, 10- and 24-h cold treatment), up to a total of 1 μg for cDNA synthesis with a QuantiTect Reverse Transcription Kit (Qiagen Inc., USA), which includes a genomic DNA removal step. The cDNAs were diluted (1:20) in nuclease-free water, and 1 μL was used for the qPCR.

Quantitative RT-PCR was conducted in a CFX384 Real-Time PCR Detection System (Bio-Rad Laboratories Inc., USA) with Maxima SYBR Green qPCR Master Mix (Thermo Scientific Inc., USA) following the manufacturers’ instructions and at a final primer concentration of 0.3 μM. The reaction conditions were as follows: 95 °C for 10 min; 40 cycles at 95 °C for 30 s, 60 °C for 30 s and 72 °C for 30 s. No template controls for any primer pair were included, and each reaction was performed in triplicate.

The evaluated sequences were similar to NAD(P)H-quinone oxidoreductase subunit H (NADH), chloroplast photosystem II 10 kDa polypeptide (PsbR), a hypothetical protein (HYPOT), ATP synthase CF0 C subunit (CF0) and indole-3-acetic acid-induced protein (ARG2-1 and ARG2-2). The glyceraldehyde-3-phosphate dehydrogenase (GAPDH) and the eukaryotic translation initiation factor (eIF2; Li et al. 2011) genes were used as reference genes, and the 0-h cold treatment and PB 217 0-h cold treatment samples were used as the controls for gene expression normalization of the combined samples and individual sample analyses, respectively. The presence of single amplicons in the PCR products was confirmed by analyzing their melting curves at temperatures ranging from 65 to 95 °C. The baseline and Cq values were automatically determined, and expression analysis was performed using CFX Manager 2.1 software (Bio-Rad Laboratories Inc., USA). All primer sequences except eIF2 are described in Online Resource 1—Table S2.

### Search for putative molecular markers

SSR mining was performed using the SciRoKo software (version 3.3; Kofler et al. 2007) with the “Perfect; MISA-mode” search mode and default settings. A sequence was defined as a microsatellite region if it exhibited the following characteristics: six repeats for dinucleotides; five repeats for trinucleotides; and four repeats for tetra-, penta- and hexanucleotides.

Contigs with a minimum of fourfold coverage were utilized for SNP mining using the CLC Genomics Workbench software (CLC bio A/S, Denmark). The minimum quality of the central base and the average quality of the surrounding bases were set at 20, and putative SNPs were annotated when the less-represented allele was present in at least two EST sequences up to a minimum frequency of 10 %. The candidate SNPs were classified according to the type of single-base substitution and visually localized into the probable exonic and untranslated (UTR) regions if the EST had a BLASTX hit.

### EST-SSR marker characterization and species transferability

PRIMER3 software (Rozen and Skaletsky 2000) was used to design EST-SSR primer pairs from the flanking sequences. The target amplicon size was set to 100–300 bp. The optimal annealing temperature was set to 60 °C, and the optimal primer length was set to 20 bp. For SSR genotyping and characterization, we used a 4300 DNA Analyzer (LI-COR Biosciences, USA), an Advance FS96 dsDNA Fragment Analyzer (Advanced Analytical Technologies Inc., USA) and 6 % denaturing polyacrylamide gels with silver staining (Creste et al. 2001).

For the analysis performed with the 4300 DNA Analyzer (LI-COR Biosciences, USA), the M13F sequence was added to the 5′ end of the forward primer of 115 primer pairs. PCR amplification was performed as described by Le Guen et al. (2011) with the following modifications: (TD1) ten amplification cycles with a 0.5 °C decrease in annealing temperature per cycle, starting at 57 °C (95 °C for 1 min, 57 °C for 30 s and 72 °C for 1 min); (TD2) ten amplification cycles with a 1 °C decrease in annealing temperature per cycle, starting at 65 °C (95 °C for 1 min, 65 °C for 30 s and 72 °C for 1 min); and (TD3) ten amplification cycles with a 1 °C decrease in annealing temperature per cycle, starting at 62 °C (95 °C for 1 min, 62 °C for 30 s and 72 °C for 1 min).

For the SSR characterization using an Advance FS96 dsDNA Fragment Analyzer (Advanced Analytical Technologies Inc., USA) and silver-stained 6 % denaturing polyacrylamide gels (Creste et al. 2001), the amplification reactions were performed as follows: denaturation at 95 °C for 3 min followed by 35 amplification cycles (95 °C for 1 min, specific annealing temperature for 45 s and 72 °C for 1 min) and a final extension step at 72 °C for 5 min. All of the amplification products were verified by 3 % agarose gel electrophoresis.

The allelic polymorphic information content (PIC) of the SSRs was calculated using the following formula:

$${\text{PIC}} = 1 - \sum\limits_{i = 1}^{n} {p_{i}^{2} } - \sum\limits_{i = 1}^{n} {\sum\limits_{j = i + 1}^{n} {2p_{i}^{2} p_{j}^{2} } }$$

where *n* is the number of alleles of the marker among the set of genotypes used for the characterization of the SSR polymorphism and *p*_*i*_ and *p*_*j*_ are the frequencies of alleles *i* and *j*, respectively. The TFPGA program (Miller 1997) was used to calculate the expected and observed heterozygosities.

### SNP marker validation and characterization

Sequences that showed similarity to known proteins were chosen for the validation of SNPs in 36 *H. brasiliensis* genotypes (Online Resource 1—Table S1). Primer pairs were designed using PRIMER3 software (Rozen and Skaletsky 2000) to validate at least one SNP present in the EST sequence. The target amplicon size depended on the position and number of putative SNP(s). The optimal annealing temperature was set to 60 °C, and the optimal primer length was set to 20 bp. PCR amplification was carried out in 20-µL reaction mixtures, each containing 25 ng of genomic DNA, 2 µM MgCl_2_, 0.2 µM dNTPs, 1× reaction buffer, 0.2 µM of each primer and 0.5 U of Pfu DNA polymerase (recombinant; Thermo Scientific Inc., USA), which was used to reduce the rate of nucleotide incorporation errors. PCR was performed using the following thermal cycling conditions: denaturation at 95 °C for 3 min followed by 35 amplification cycles (95 °C for 30 s, specific annealing temperature for 30 s and 72 °C for 2 or 3 min) with a final extension step at 72 °C for 10 min. The PCR products were verified by 1.5 % agarose gel electrophoresis.

For sequencing, the amplicons were purified using a solution containing 20 % PEG8000 (w/v) and 2.5 M NaCl solution. The sequencing of the amplicons was carried out bidirectionally (forward and reverse) in a 10-μL reaction mixture using the Big Dye Terminator 3.1 Cycle Sequencing kit (Applied Biosystems Inc., USA). The sequencing reactions were analyzed using a 3500XL DNA ABI PRISM Automatic Sequencer (Life Technologies Corporation, USA). The chromatograms were aligned to the reference sequence using ChromasPro 1.5 software, and the SNPs were identified as overlapping nucleotide peaks. The expected and observed heterozygosities of the polymorphic positions and their PIC values were calculated using the same methods used for EST-SSR marker characterization.

## Results and discussion

### Library construction, characterization, sequencing and contig assembly

To develop gene-targeted molecular markers, six standard cDNA libraries were constructed from the leaves of cold-stressed and panel, latex and leaf tissues of different rubber tree clones (see “Methods”). Colony PCR revealed that the cDNA inserts ranged from 200 bp to 2.8 kb in length. A total of 10,080 clones of the cDNA libraries, consisting of all the clones from the cold-stressed leaf libraries (cold-6 h and cold-10 h: 1,824 clones each; cold-24 h: 2,496 clones) and randomly chosen clones from the panel, latex and leaf libraries (1,536, 1,632 and 768 clones, respectively), were subjected to sequencing. After removing the vector, adaptor, low-quality and short sequences (<150 bp) as well as all possible contaminating sequences, a total of 8,263 (82 %) EST sequences were generated, with an average length of 664 bp. The percentage of redundant sequences was approximately 41 %.

CLC Genomics Workbench 4 (CLC bio A/S, Denmark) and Chromas Pro 1.5 (Technelysium Pty Ltd., Australia) were used to assemble the ESTs, generating 5,025 unigenes composed of 816 contigs and 4,209 singletons. The majority of these sequences (3,640; 72.4 %) had lengths of between 200 bp and 1 kb. The average length of the unigene sequences was 715 bp, which is longer than the *Hevea brasiliensis* ESTs from the panel (Li et al. 2012), leaf and latex (Xia et al. 2011) sequences obtained from RNA-seq experiments and longer than the ESTs obtained from *M. ulei*-infected leaves (Garcia et al. 2011). The contigs were formed from between two (413 contigs) and 162 (one contig) reads, and the average number of reads per contig was 4.97 (Online Resource 1—Table S3). Among the 5,025 sequences, 4,991 (99.3 %) contained ORFs, and after read assembly, the sequence redundancy decreased to 1.5 %.

### The most highly represented genes in the EST sequences

EST assembly was performed for each library prior to the assembly of all sequences, and the number of reads present in the generated contigs was evaluated. In the cold-6 h library, two contigs formed by 33 reads each were the most highly expressed based on the number of ESTs in a contig. Both contigs exhibited similarity to proteins related to photosynthesis (NAD(P)H-quinone oxidoreductase subunit H, *e*-value 7e^−148^, and chloroplast photosystem II 10 kDa polypeptide (PsbR), *e*-value 2e^−65^). A hypothetical protein (*e*-value 2.83e^−14^) was the most highly expressed sequence in the cold-10 h library (36 reads) and the second most highly expressed in the cold-24 h library (37 reads) and was highly represented in the leaf library (63 reads), which suggests that this transcript might be important for the leaf tissue, although no probable function has been described for this transcript. The second most highly represented sequence in the cold-10 h library was identified by 25 reads and was similar to ATP synthase CF0 C subunit (*e*-value 3e^−28^), which is also involved in photosynthesis.

The cold-24 h library also presented highly represented (≥20 reads) sequences similar to proteins involved in photosynthesis, but the most highly expressed sequence in this library (with 44 reads) was similar to indole-3-acetic acid-induced protein (ARG2; *e*-value 7e^−31^). There was also a second contig formed by seven reads that matched the same protein. This sequence was also present at a very low frequency in the cold-6 h, cold-10 h and panel libraries (two, four and two reads, respectively). This protein is associated with the stress response in plants (Yamamoto et al. 1992; Seki et al. 2001).

Sequences similar to the rubber elongation factor protein (REF; *e*-value 6e^−91^; 88 reads) and pro-hevein (54 reads; *e*-value 2e^−144^) were the most highly represented ESTs in the latex library. REF and small rubber particle protein (SRPP), which was represented by two contigs composed of 36 and 26 reads, are believed to be involved in latex biosynthesis (Dennis and Light 1989; Oh et al. 1999) and are highly expressed in latex and laticifers (Ko et al. 2003; Chow et al. 2007). Pro-hevein is believed to be involved in the defense response because it is able to bind to chitin and inhibit fungal growth (Van Parijs et al. 1991); pro-hevein is also abundant in latex and laticifers (Ko et al. 2003; Chow et al. 2007). The panel library sequences seemed to be less redundant than the sequences from the other libraries because most of the contigs of the panel library were formed by fewer than seven reads. The most represented sequences in this library presented similarity to non-specific lipid transfer protein (17 reads; *e*-value 1e^−40^) and metallothionein 3-like protein (15 reads; *e*-value 1e^−24^).

The contigs were also analyzed after the assembly of all ESTs. Because a majority of the ESTs originated from the leaf tissues, reads exhibiting similarity to chloroplast sequences, such as the proteins of photosystems I and II, were highly abundant, as expected. These reads constituted 13 of the 27 most highly expressed unigenes, considering the number of ESTs in a contig (≥20 reads). These 27 unigenes accounted for 15.1 % of the 8,263 sequences obtained. The following contigs did not show similarity to any protein in the GenBank database but were similar to other EST sequences: contig 366, which had 47 reads from the cold-stressed leaf libraries; contig 42; and contig 142. Sequences similar to REF, SRPP and pro-hevein were also highly represented, mostly due to the sequences from the latex library. All of the 27 most expressed unigenes are described in Online Resource 1—Table S4.

### Expression analysis of the most highly represented genes in the cold-stressed leaf libraries

**Fig. 1:**
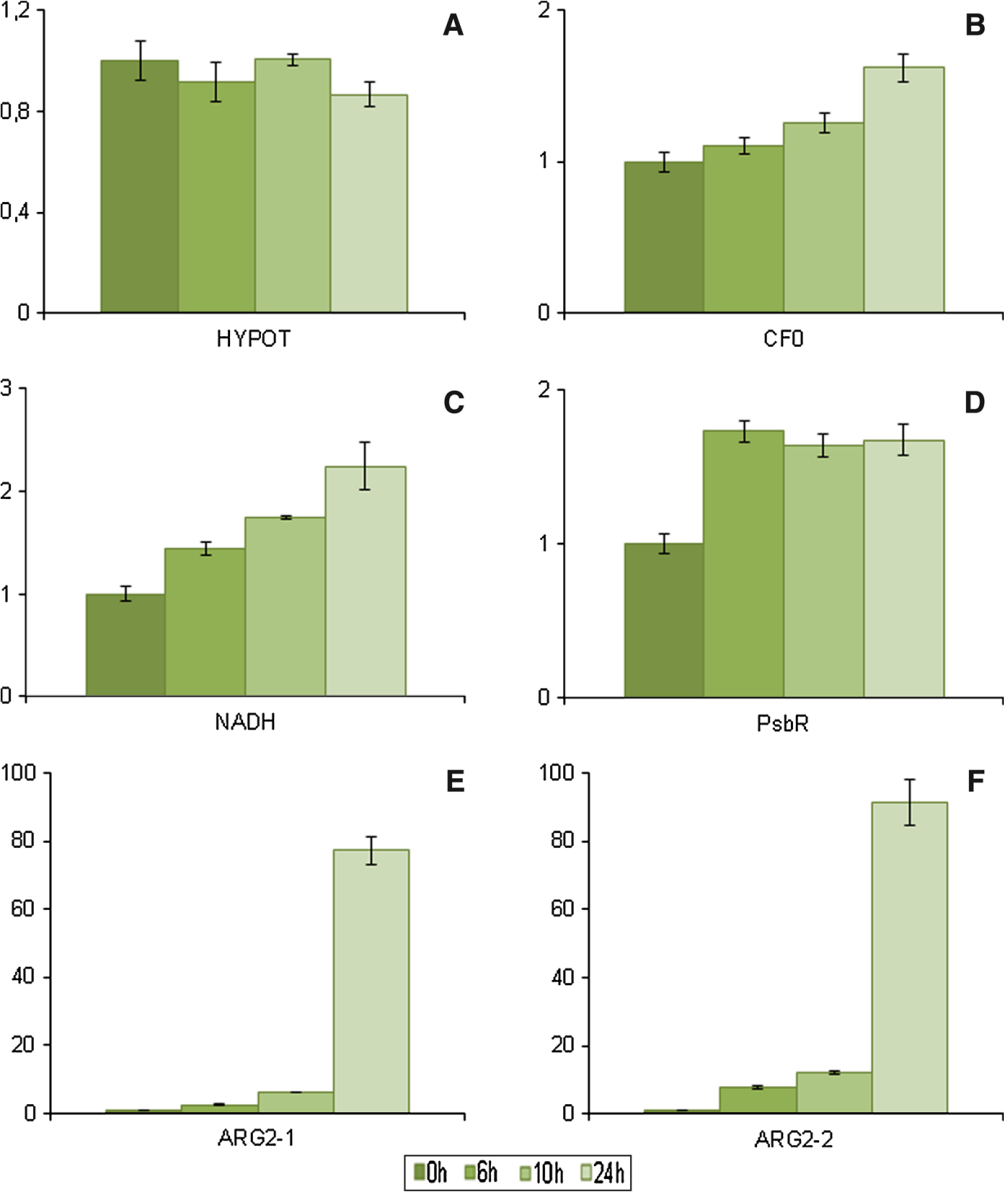
Expression analysis of the most highly represented sequences in the cold-stressed leaf libraries **a** hypothetical protein (HYPOT), **b** ATP synthase CF0 C subunit (CF0), **c** NAD(P)H-quinone oxidoreductase subunit H (NADH), **d** chloroplast photosystem II 10 kDa polypeptide (PsbR), **e** and **f** indole-3-acetic acid-induced proteins (ARG2-1 and ARG2-2)

Quantitative RT-PCR analysis was performed to examine the expression of the sequences that presented the highest number of reads in each of the cold-stressed leaf libraries (6, 10 and 24 h) plus the additional sequence also similar to the ARG2 protein. The expression of the hypothetical protein did not differ among the combined samples (Fig. 1a); however, there was a 5.4-fold decrease in the expression of the clone GT 1 6 h sample (Fig. S1A). Clone GT 1 appeared to maintain a high level of expression of this sequence when compared to the other clones, and low temperature had an effect on its transcription. Because no function has been assigned to this protein, the processes that it may be involved in remain to be investigated. All other sequences evaluated in the combined samples presented some level of up-regulation. ATP synthase CF0 C subunit is a membrane component of the chloroplast ATP synthase complex (Seelert et al. 2000). A 1.6-fold increase in its expression at 24-h cold exposure was observed in comparison with the 0 h sample (Fig. 1b). Chilling-stress impairs the function of the ATP synthase complex through the production of reactive oxygen species (ROS; Prasad et al. 1994; Buchert et al. 2012). The increase in the expression of this subunit may be due to the required replenishment of novel ATP synthase complexes in chloroplasts. Clones PB 217 and PR 255 presented an increase in the expression of this sequence from the 10 and 6 h samples, respectively. There was a 1.8-fold decrease in its expression in the GT 1 sample at 6 h. The expression level remained similar thenceforth, and clone IAN 873 did not show a significant difference among samples (Fig. S1B). Clones GT 1 and IAN 873 may exhibit a better protection of their photosynthetic apparatus against oxidative stress than clones PB 217 and PR 255. This protection may also be related to their tolerance to low temperatures.

The NAD(P)H-quinone oxidoreductase subunit H showed a gradual increase in its expression among all combined samples (1.4-fold in 6 h, 1.7-fold in 10 h and 2.2-fold in 24 h; Fig. 1c) and was also up-regulated in the individual clone samples (Fig. S1C). The NAD(P)H-quinone oxidoreductase complex seems to be important for cold-stress response. In tobacco, mutants with deleted subunits of this complex present a more severe phenotype under chilling stress than wild-type plants (Li et al. 2004; Wang et al. 2006). This expression increase might be a response to cope with the low temperature.

PsbR protein is important for the proper assembly of the oxygen-evolving complex of the photosystem II (PSII) complex (Suorsa et al. 2006) and demonstrated a 1.7-fold increase in its expression in leaf tissues after 6 h low temperature exposure, which was maintained in the other combined samples (Fig. 1d). A similar pattern was observed in the clones’ individual samples, except for IAN 873, which presented a 1.6-fold increase in expression in the 6 h sample, whereas the 10 and 24 h samples showed the same expression level as the 0 h sample (Fig. S1D). *Arabidopsis thaliana* *PsbR* mutants present higher PSII excitation pressure than wild-type plants (Suorsa et al. 2006), which is also caused by low temperature (Huner et al. 1998). The increase in *PsbR* expression after chilling stress may be related to the photoprotection of the PSII complex.

ARG2 is a late embryogenesis abundant (LEA)-type protein, a group of hydrophilic proteins present in a wide range of plant species that are induced by water deficit caused by desiccation, cold and osmotic stress (Wang et al. 2003). Two sequences that presented similarity to the ARG2 protein were evaluated by qPCR: ARG2-1 (24 h—44 reads), for which SNP markers were developed (see below), and ARG2-2 (24 h—7 reads); both sequences were up-regulated. ARG2-1, in comparison with the 0 h combined sample, presented an increase in expression of 2.6-fold in the 6 h sample, 6.3-fold in the 10 h sample and 77-fold in the 24 h sample (Fig. 1e). An analysis of the individual samples demonstrated a large difference in the up-regulation of this sequence between the PB 217 clone 24 h sample (38-fold) and the other clones’ 24 h samples (>100-fold) (Fig. S1E). The expression of ARG2-2 was up-regulated in the combined samples by 7.7-fold, 12-fold and 91-fold after 6, 10 and 24 h of cold exposure (Fig. 1f), respectively. This sequence was also less up-regulated in the PB 217 clone 24 h sample (80-fold) when compared to the other clones’ 24 h samples. Clone PR 255 presented a 395-fold expression increase in ARG2-2 after 24 h of cold exposure, while GT 1 showed a 249-fold up-regulation and IAN 873 presented a 171-fold increase in ARG2-2 expression (Fig. S1F).

*ARG2* transcription increased in the presence of indole-3-acetic acid (IAA; Yamamoto et al. 1992). It had been previously observed that the IAA levels increase in *A. thaliana* (Gray et al. 1998) and rice (Du et al. 2013) under temperature stress. Our results suggest that these sequences were up-regulated due to the prolonged exposure of the rubber tree clones to low temperatures. Similarly, the increase in the expression of these sequences might be due to increased levels of IAA in the clones. In addition, the higher expression of these sequences in clones PR 255, GT 1 and IAN 873 might be related to their better tolerance to low temperatures when compared to clone PB 217. These results may require further detailed analysis because only one individual of each clone was evaluated. Nevertheless, these data demonstrate that the individuals used in our work exhibit different responses to cold stress.

### Functional annotation

To identify unigenes that were likely to encode proteins with known functions, sequences were subjected to BLASTX analysis against the GenBank non-redundant protein database using BLAST2GO software (Conesa et al. 2005). A total of 3,456 (68.8 %) unigenes showed significant similarity (*e*-value <1e^−06^) to at least one unknown, hypothetical or expressed protein, and 3,404 (98.5 %) unigenes had *e*-values less than 1e^−10^. Proteins from *Ricinus communis* accounted for the majority of BLASTX hits for these sequences (1,771), followed by proteins from *Populus trichocarpa* (709), *Vitis vinifera* (240) and *H. brasiliensis* (236) (Online Resource 1—Fig. S2). *R. communis* also belongs to the Euphorbiaceae family, and the GenBank database contains information on a large number of *R. communis* and *P. trichocarpa* proteins (68,409 and 104,560 proteins, respectively, as of November 2013); this abundance of data may explain the similarity between and number of hits for *R. communis* and *P. trichocarpa* proteins. In comparison, the GenBank database contains fewer rubber tree proteins (1,120), which may have led to the limited number of *H. brasiliensis* protein hits. The remaining 1,569 (31.2 %) sequences that did not show a significant similarity to any protein in the database and therefore could not be annotated were subjected to BLASTN analysis (*e*-value <1e^−06^). Of these, 657 ESTs were similar to ESTs that are present in the GenBank database, leaving 912 (18.2 %) sequences with no hits. The sequence length may affect the annotation success of reads. Among the sequences without hits, 358 ESTs had lengths shorter than 400 bp, accounting for 41.7 % of the analyzed unigenes. In contrast, 9.6 % (196) of the sequences longer than 800 bp did not match any sequence in the GenBank database. These ESTs may be considered to be novel or specific transcripts of *H. brasiliensis*.

**Fig. 2:**
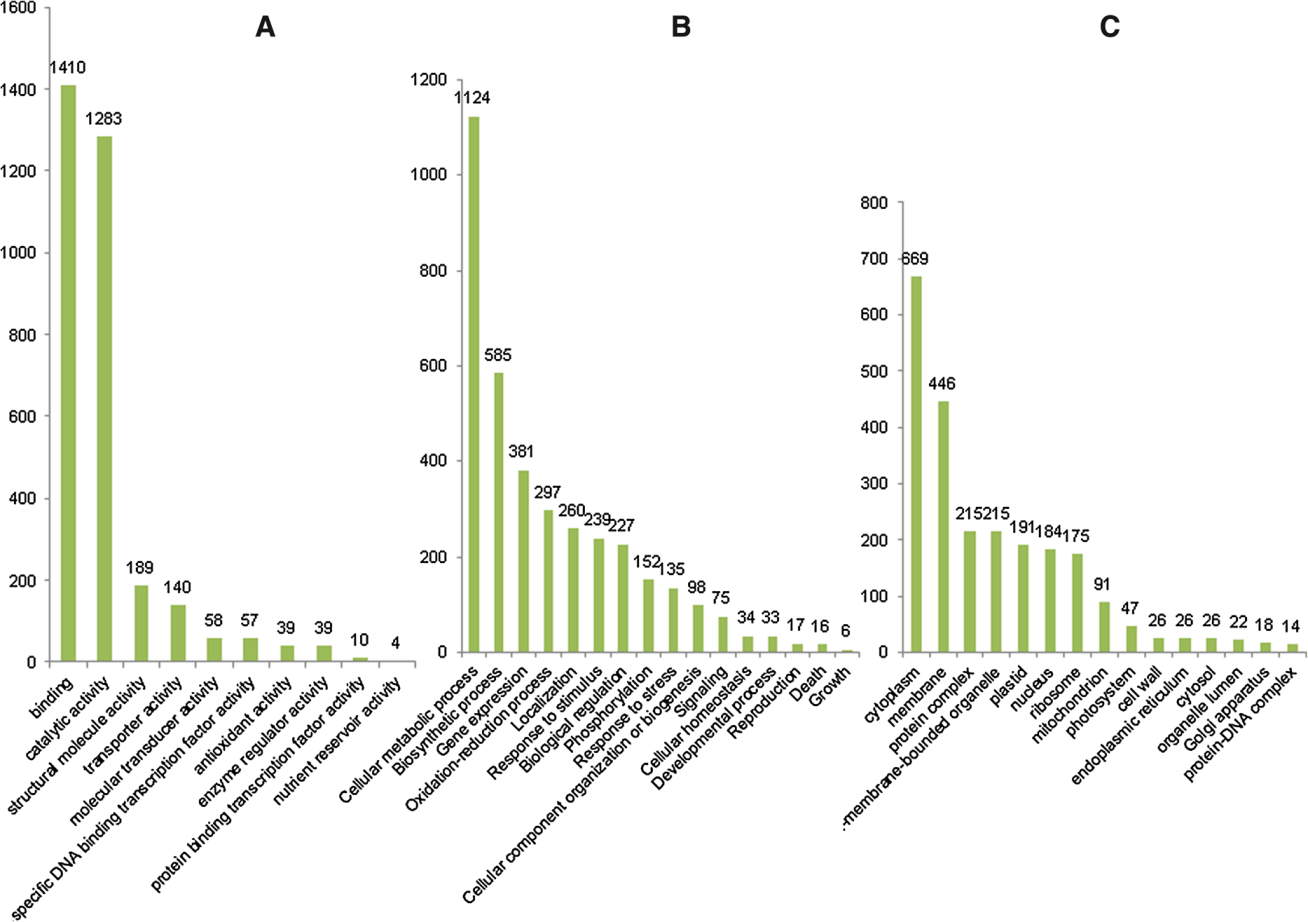
Functional category distribution of the annotated unigenes **a** molecular function (MF), **b** biological process (BP) and **c** cellular component (CC)

A functional classification of the unigenes was performed according to the GO database using the BLAST2GO program. The terms were organized into three main categories: molecular function (MF), biological process (BP) and cellular component (CC). Of the 3,456 sequences analyzed, 2,503 (72.4 %) were annotated with 8,232 terms, including 3,867 MFs, 2,743 BPs and 1,622 CCs. In the MF category, binding (1,410; 36.5 %) and catalytic activity (1,283; 33.2 %) were the largest categories, followed by structural molecule activity (189; 4.9 %; Fig. 2a). Cellular metabolic process (1,124; 41.0 %), biosynthetic process (585; 21.3 %) and gene expression (381; 13.9 %) were the most highly represented categories in the BP category (Fig. 2b). In the CC category, the most represented categories were cytoplasm (669; 41.2 %), membrane (446; 27.5 %) and the protein complex and non-membrane-bounded organelle, which shared the same number of sequences (215; 13.3 %; Fig. 2c).

In addition to the GO terms, 1,050 enzyme commission (EC) numbers were attributed to 910 unigenes. To establish associations with biological pathways, KEGG pathway analysis was used to map the 1,050 EC numbers into 116 KEGG pathways. Some sequences were mapped to more than one pathway. The majority of the mapped unigenes were related to carbohydrate metabolism pathways, and starch and sucrose metabolism (48 sequences, 22 enzymes) and glycolysis/gluconeogenesis (36 sequences, 16 enzymes) were the most highly represented in this group. The purine metabolism pathway had the largest number of unigenes (51), representing 18 enzymes. Other highly represented categories included carbon fixation in photosynthetic organisms (45 sequences, 18 enzymes), in which the majority of the mapped sequences originated from libraries of leaf tissues; nitrogen metabolism (40, 12); and oxidative phosphorylation (34, 7). Enzymes involved in secondary metabolism-related pathways were also identified; a majority of these were involved in phenylpropanoid biosynthesis (29, 4) and flavonoid biosynthesis (26, 13). Sixteen of the sequences that were mapped to the phenylpropanoid biosynthesis pathway were annotated as lactoperoxidase (EC 1.11.1.7), which is involved in lignin biosynthesis and stress response (Dixon and Paiva 1995), and the majority of the sequences mapped to the flavonoid biosynthesis pathway were identified from the panel library. The 16 most represented pathways (≥15 unigenes) are listed in Online Resource 1—Table S5. Sequences that showed similarity to genes involved in the 2-C-methyl-d-erythritol 4-phosphate (MEP) pathway (six sequences, five enzymes; Sando et al. 2008), which is believed to be one of the metabolic pathways involved in rubber biosynthesis, were also identified.

### The characterization and development of the EST-derived SSR markers

SciRoKo software (version 3.3; Kofler et al. 2007) was used in MISA mode to identify microsatellite regions in the 5,025 sequences analyzed. A total of 588 microsatellite regions were identified, and 527 unigenes (10.5 %) contained at least one of the defined motifs (di-, tri-, tetra-, penta- or hexanucleotides). Of these sequences, 58 contained more than one SSR region. Nineteen microsatellites were present as compound SSRs; however, each motif was considered as a single repeat. On average, one SSR was found per 6.1 kb in the 3,578,774 bp of EST sequences that were searched, which is higher than the frequency reported for rice (one SSR per 3.4 kb; Cardle et al. 2000) and castor bean (1.23 kb; Pranavi et al. 2011) and similar to that of *Jatropha curcas* (6.0 kb; Yadav et al. 2011) but lower than that of bread wheat (9.2 kb; Gupta et al. 2003), soybean (7.4 kb), maize (8.1 kb), tomato (11.1 kb), poplar (14.0 kb) and cotton (20.0 kb; Cardle et al. 2000). The proportion of SSR unit sizes was not evenly distributed. Among the 588 SSR regions found, dinucleotide (302, 51.4 %) motifs were the most frequent, followed by tri- (205, 34.9 %), tetra- (39, 6.6 %), penta- (27, 4.6 %) and hexanucleotide motifs (15, 2.6 %). Several previous EST studies have shown that trinucleotide repeats are the most abundant microsatellite type in the expressed sequences of many plants (Cardle et al. 2000; Gupta et al. 2003; Clepet et al. 2011), whereas other studies showed that dinucleotide motifs were more frequent (Pranavi et al. 2011; Yadav et al. 2011). In our analysis, dinucleotide repeats were the most highly represented SSR motif in the unigenes, in agreement with previous studies of rubber tree ESTs (Feng et al. 2009; Triwitayakorn et al. 2011; Li et al. 2012). Nevertheless, these differences in the distribution and frequency of SSR regions among the different crops may be due to the SSR search criteria, the number of total ESTs and bases searched and the software tools used (Varshney et al. 2005a), making a direct comparison of the abundance and frequency of SSR motifs difficult.

The most frequent type of dinucleotide motif was AG/TC (167, 55.3 %), followed by AT/TA (107, 35.4 %) and AC/GT (27, 8.9 %). The AAG/TTC motif (82, 40.0 %) was the most common trinucleotide repeat (Online Resource 1—Table S6). Among the dinucleotide repeats, the GC/CG motif was identified only once. This repeat motif appears to be rare in most plant ESTs; GC repeats were found at a very low frequency in previous studies (Pranavi et al. 2011; Yadav et al. 2011; Clepet et al. 2011), including those involving the rubber tree (Feng et al. 2009; Triwitayakorn et al. 2011; Li et al. 2012). This low frequency of CG repeats and CCG repeats in EST sequences may be due to the methylation of CpG sequences, which may inhibit transcription (Lister et al. 2008).

SSR-containing sequences that showed similarity to proteins in the GenBank database were preferentially chosen for the development of microsatellite markers. A total of 211 primer pairs were designed based on 202 SSR-containing sequences; of these primer pairs, 18 were designed from nine ESTs bearing two different SSR regions. These sequences were subjected to a BLASTN (*e*-value <1e^−06^) search against the GenBank database to remove possible redundancies with published SSRs. Only one sequence, bearing a dinucleotide motif, was identical to a previously published locus, and this sequence was thus removed from this study. Of the 210 non-redundant primer pairs, 83, 97, 19, 3 and 8 primer pairs were designed to amplify di-, tri-, tetra-, penta- and hexanucleotide motifs, respectively. The M13 tail was added to the 5′ end of 115 forward primers for the fluorescence analysis of these loci using a 4300 DNA Analyzer (LI-COR Biosciences, USA). The other 95 primer pairs were analyzed via silver-stained 6 % acrylamide gel electrophoresis (Creste et al. 2001) or capillary electrophoresis using an Advance FS96 dsDNA Fragment Analyzer (Advanced Analytical Technologies Inc., USA). After the primers for fluorescence analysis were tested in different touchdown programs and the primers for acrylamide and capillary electrophoresis analysis were tested at different annealing temperatures, 196 primer pairs (93.3 %) produced amplicons, as shown by 3 % agarose gel electrophoresis. Of the 196 working primer pairs, 178 amplified PCR products of the expected sizes and 18 produced larger PCR products than expected. Of these 18 products, ten were amplicons that ranged from 500 bp to 1 kb. Because the primers were designed based on expressed sequences and genomic DNA was used for amplification, the existence of these larger PCR products suggests the presence of intronic regions in the genomic sequences.

Eighteen *H. brasiliensis* genotypes (Online Resource 1—Table S1) were used to assess the polymorphism of the 186 primer pairs that produced amplicons smaller than 400 bp. Seventeen of these 186 primer pairs showed non-specific amplification and could not be evaluated; thus, 169 primer pairs were analyzed, and 137 were polymorphic among the 18 genotypes tested. Among the 161 ESTs used to design these primer pairs, 141 were annotated as known or uncharacterized proteins; thus, 147 SSR loci (87.0 %) may be associated with possible functional genes.

One of the polymorphic primer pairs (EHBp-23) amplified two distinct polymorphic regions, resulting in 138 polymorphic loci. The EST used to design this primer pair was obtained from the panel library and showed similarity at the nucleotide sequence level to the protein aquaporin, which is involved in water transport and belongs to the large major intrinsic protein (MIP) family of transmembrane channels. Several genes encoding aquaporins have been discovered in plants (Chaumont et al. 2005). Because aquaporins are a highly conserved group of proteins, the two loci amplified may represent different genes that encode aquaporins in *H. brasiliensis*.

The expected (*H*_*e*_) and observed (*H*_*o*_) heterozygosities and polymorphic information content (PIC) values could only be calculated for 136 of the 138 polymorphic loci because two loci contained duplicated alleles in several genotypes (see below). *H*_*e*_ and *H*_*o*_ ranged from 0.0556 to 0.89 (average 0.4648) and 0–1 (average 0.3622), respectively, and the mean number of alleles was 3.7 (2–10 alleles). Nevertheless, at several loci, an exclusive allele was present in only one or two of the genotypes tested, and in most cases, clones RRIM 809, RRIC 100, IAC 306 or RRII105 were the bearers of this allele. Expressed regions show a greater level of DNA sequence conservation (Varshney et al. 2005a), which explains both the lower number of alleles observed in EST-SSRs compared to genomic SSR markers (Souza et al. 2009; Mantello et al. 2012) and the presence of rare alleles. The PIC values ranged from 0.0526 to 0.8512, with an average of 0.4036, indicating that this group of EST-SSR markers presents a moderate level of informativeness. Although this group of markers presented a low to moderate level of polymorphism, these markers will be useful for genetic mapping, determining the linkage between markers and genes for important traits, QTL mapping, marker-assisted selection and functional analysis of candidate genes in the rubber tree, among other information. All 169 primer pairs are fully described in Online Resource 2.

The two loci that could not be analyzed exhibited duplicated alleles in several rubber tree accessions. Of the 18 genotypes of *H. brasiliensis* used in this study, nine showed duplicated alleles for the SSR region amplified by primer pair EHBc-103 and 15 contained duplicated alleles for the region amplified by primer pair EHBp-27, resulting in six and ten different alleles, respectively. Plants of the *Hevea* genus exhibit diploid behavior, mainly forming bivalents during meiosis (Bouharmont 1960; Majumder 1964; Ong 1975); however, cytogenetics studies have revealed two loci on different chromosomes bearing the same rDNA sequence, suggesting a possible allotetraploid origin (Leitch et al. 1998). Although the species has a diploid genome, molecular marker analyses revealed locus duplication in *H. brasiliensis* (Lespinasse et al. 2000; Mantello et al. 2012). These duplicated loci are likely due to the allotetraploid origin of the species (Lespinasse et al. 2000). The EST sequences used to design these primer pairs showed similarity to proteins that mapped to different chromosomes in *Glycine max*, a diploidized tetraploid (Shoemaker et al. 1996). Although no potential ancestor has yet been described for the rubber tree (Leitch et al. 1998), our results support the hypothesis of a polyploid origin followed by a diploidization event.

### Cross-species transferability

The transferability of the 169 primer pairs to other *Hevea* species was also evaluated; 167 (98.8 %) primers successfully produced amplicons in at least one of the six species tested: 164 (97.0 %) were amplified in *H. guianensis*, 158 (93.5 %) in *H. nitida* and *H. benthamiana*, 157 (92.9 %) in *H. rigidifolia*, 156 (92.3 %) in *H. pauciflora* and 148 (87.6 %) in *H. camargoana* (Online Resource 2). In addition to this high transferability, the number of alleles per locus increased to 5.6 when compared with *H. brasiliensis* alone, revealing the presence of novel alleles. As expected, the transferability rates of the EST-SSR markers were higher than those of the genomic SSR markers (Varshney et al. 2005a, b; Feng et al. 2009; Mantello et al. 2012). The EST-SSR markers are likely related to gene units, and as such, their potential for inter-specific transferability is greater (Gupta et al. 2003; Andersen and Lübberstedt 2003; Varshney et al. 2005a). Our results indicate that the SSR flanking regions are conserved among the *Hevea* species, consistent with other studies (Feng et al. 2009; Souza et al. 2009; Mantello et al. 2012). Additionally, the *Hevea* genus is considered to be a complex of species without genetic reproductive barriers between them. This characteristic of the genus has been used in rubber tree breeding programs for inter-specific crosses, mostly with the aim of developing clones that are tolerant or resistant to SALB (Gonçalves and Fontes 2012). These loci could be used to follow gene introgressions in the resulting hybrids and to provide markers for comparative mapping and for population structure and genetic analyses of these species.

As described previously (Souza et al. 2009; Mantello et al. 2012), the other *Hevea* species also presented duplicated alleles. The primer pair EHBc-103 amplified more than two alleles in all species, except *H. pauciflora*, and *H. rigidifolia* was the only one that did not have more than two alleles in the region amplified by EHBp-27. Taken together with previous results, our results suggest that the other *Hevea* species analyzed also have duplicated loci in their genomes.

### Development and characterization of the SNP markers

**Fig. 3:**
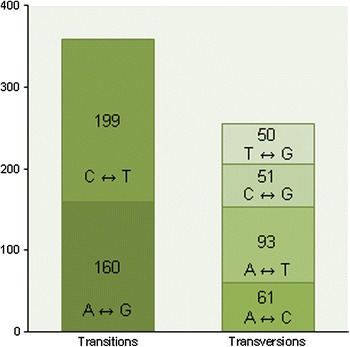
Distribution of the putative SNPs identified in this study

For SNP discovery, CLC Genomics Workbench software (CLC Bio A/S) was used to assemble the 8,263 EST sequences obtained, generating 816 contigs. From these contigs, 121 (composed of 2,429 reads with a total length of 109,512 bp) had coverage equal to or greater than four and were therefore analyzed for SNP identification. A total of 614 putative SNPs (359 transitions and 255 transversions) were identified in 104 contigs. Transitions are the most common SNP variant in several plants (Wu et al. 2008; Novaes et al. 2008; Clepet et al. 2011; Gaur et al. 2012), including the rubber tree (Pootakham et al. 2011). The most frequent variation was C ↔ T, and the least frequent variation was G ↔ T (Fig. 3). On average, an SNP was identified every 178 bp, which is comparable to the rate observed in other plant species, such as almond (1 SNP/114 bp; Wu et al. 2008) and *Eucalyptus grandis* (1 SNP/192 bp; Novaes et al. 2008). However, the SNP frequency in *H. brasiliensis* was reported to be approximately eightfold higher (1 SNP/1.5 kb; Pootakham et al. 2011) than the frequency determined in the present work. The sequences of two clones that shared a parent were analyzed in the previous study (Pootakham et al. 2011), whereas in the current work, ESTs from six clones with different parents were used for assembly and SNP mining. Most likely, this difference in SNP frequency was due to the different numbers and genotypes of *H. brasiliensis* used. Most of the putative SNPs identified (376, 61.2 %) were located in exonic regions, whereas 45 (7.3 %) were identified in 5′ UTRs, 150 (24.4 %) were in 3′ UTRs and 43 (7.0 %) were located in “no-hit” sequences (Table 1).

**Table 1:** Summary statistics of the SNP analysis

	Numbers
Number of contigs used	121
Number of contigs with putative SNPs	104
Total contig length	109,512 bp
Average number of reads per contig	19.9
Number of identified putative SNPs	614
Average number of SNPs per contig	5.03
SNP frequency	1/178 bp
Localization	
5′ UTR	45
Exon	376
3′ UTR	150
No-hit	43

Of the 104 contigs in which SNPs were identified, 16 sequences that showed similarity to proteins involved in the stress response, rubber biosynthesis and developmental processes were chosen to validate an SNP subset. Sixteen primer pairs flanking 61 putative SNPs were designed with expected amplicon sizes of approximately 200–800 bp. Thirteen primer pairs amplified products in the 36 *H. brasiliensis* genotypes used (Online Resource 1—Table S1), and the amplicons observed by 1.5 % agarose gel electrophoresis ranged from 250 bp to 1.5 kb, with five loci showing PCR products that were longer than the expected length. Sequencing and a comparison of the chromatograms to the original sequence revealed the presence of intronic regions in these loci; the smallest intron was approximately 80 bp in length, and the longest was approximately 1.1 kb. A total of 46 putative SNPs were evaluated by visual inspection of overlapping nucleotide peaks in the chromatograms, and 43 positions (91.5 %) were validated in the 36 genotypes used. The majority of these polymorphic positions (23 SNPs) were located in probable 3′ UTRs, 18 were in exonic regions (12 non-synonymous SNPs and six synonymous SNPs), and one was located in a probable 5′ UTR. One of the non-synonymous polymorphic positions (Hb-SNP2-624) was not a true SNP but, rather, an 18-bp duplication that was considered an SNP in the CLC Genomics Workbench software alignment analysis. In the predicted translation, this duplication causes a repetition of six amino acids in the protein sequence.

The 43 polymorphic positions presented *H*_*e*_ values ranging from 0.0294 to 0.5056, with an average of 0.3566. *H*_*o*_ varied between 0 and 0.7941, with an average of 0.3256. The average PIC value was 0.2807, and the PIC values ranged from 0.0286 to 0.3742 (Table 2); these markers were therefore less informative than the EST-SSR markers developed in this work. This difference between SNP and SSR markers was also reported in other species (Jones et al. 2007; Emanuelli et al. 2013). Although SNPs are the most abundant variation found in plant genomes, they are usually limited to two alleles per locus, even when considering that a SNP locus theoretically has four different alleles. This limitation can be overcome by using multiple SNP loci to construct haplotypes, which may increase the genetic diversity and information content of these markers (Rafalski 2002; Jones et al. 2007; Emanuelli et al. 2013). Nine of the 13 loci analyzed here contain two or more SNPs in their sequence, and these SNPs together can be considered a haplotype for the locus, thereby compensating for the low informativeness of a single SNP.

**Table 2:** Validated and characterized SNP markers in the rubber tree

Name	*H*_*e*_	*H*_*o*_	PIC	BLASTX hit	Primer sequence (5′–3′)	Expected length (bp)	Observed length (bp)	Ta (°C)
Hb-SNP1-292	0.4917	0.5882	0.3671	Copper chaperone (4e^−37^) [*Populus alba* × *Populus glandulosa*]	F: TGATTTGAAGGAGCAAAAGG R: GGCATACGACCATAAAGCAC	353	~350	60
Hb-SNP1-349	0.4860	0.5588	0.3642					
Hb-SNP1-362	0.1925	0.2121	0.1716					
Hb-SNP1-455	0.3566	0.3939	0.2896					
Hb-SNP1-459	0.4909	0.5758	0.3633					
Hb-SNP1-469	0.4909	0.5758	0.3633					
Hb-SNP2-497	0.5034	0.2581	0.3726	Membrane steroid-binding protein (2e^−86^) [*Arabidopsis thaliana*]	F: ATGGACCTGGTGGACCTTAT R: CACCAAGTACATGCATCCAA	425	~430	61.6
Hb-SNP2-569	0.4779	0.3939	0.3599					
Hb-SNP2-624	0.5055	0.1111	0.3742					
Hb-SNP3-531	0.4571	0.6857	0.3491	Class IV endochitinase (7e^−94^) [*Vitis vinifera*]	F: TTCTAAACGGGAAGTTGCTG	512	~600	63.2
Hb-SNP3-535	0.486	0.7941	0.3642		R: ATTGGCGTACGTGCATTTAT			
Hb-SNP4-387	0.487	0.5714	0.3648	NAC domain protein (1e^−65^) [*Populus trichocarpa*]	F: TTCAGTACCGAAGTTGCACA R: AACCCACCCTTAAAACTACCA	432	~430	60
Hb-SNP4-572	0.5056	0.5429	0.3742					
Hb-SNP4-590	0.5004	0.4118	0.3715					
Hb-SNP5-458	0.4539	0.25	0.3457	Lipid transfer protein precursor (4e^−47^) [*Gossypium hirsutum*]	F: GCTTGAAAAGCTCTGCTGCT R: TGGGCTCTCTAACACCCATT	221	~400	63.2
Hb-SNP6-84	0.4539	0.25	0.3457	Pro-hevein (5e^−145^) [*H. brasiliensis*]	F: AATTGGGAAGAAATGGGAAG R: TGGCTCAAATGCCATTATTT	804	~880	60
Hb-SNP6-452	0.4539	0.25	0.3457					
Hb-SNP6-774	0.3398	0.2813	0.2957					
Hb-SNP7-167	0.3566	0.3333	0.2896	Indole-3-acetic acid-induced protein ARG2, putative (1e^−31^) [*Ricinus communis*]	F: GCTTCTTCCTTCCTTGTTCC R: TTTCATTCACAAGCTCAGCA	696	~700	60
Hb-SNP7-251	0.0294	0.0294	0.0286					
Hb-SNP7-273	0.4917	0.6471	0.3671					
Hb-SNP7-544	0.3951	0.4706	0.3135					
Hb-SNP7-556	0.3951	0.4706	0.3135					
Hb-SNP7-562	0.4087	0.4412	0.3216					
Hb-SNP7-586	0.3951	0.4706	0.3135					
Hb-SNP8-475	0.1874	0.2059	0.1676	GDP-L-galactose phosphorylase (3e^−131^) [*Malpighia glabra*]	F: AAGCTCTTGGGGAAGTGAGT R: CAAGTCCTGAGCATCGTTCT	242	~250	63.2
Hb-SNP9-720	0.1549	0.1667	0.1411	Rubber elongation factor (5e^−100^) [*H. brasiliensis*]	F: GCATTGTTCCTCCAATTGTC R: TTGGCCATTTATTCCCATTA	308	~300	60
Hb-SNP10-149	0.3176	0.12	0.289	Major allergen Pru ar, putative (6e^−71^) [*Ricinus communis*]	F: AAATTTTTGTTTAGACTCGCTCT R: AAGCCATCATGGGTGTTTTA	832	~900	57.5
Hb-SNP10-152	0.1502	0	0.1364					
Hb-SNP10-221	0.1502	0	0.1364					
Hb-SNP10-267	0.1448	0	0.1319					
Hb-SNP10-302	0.1448	0	0.1319					
Hb-SNP10-332	0.1448	0	0.1319					
Hb-SNP11-60	0.4329	0.3478	0.3338	Small rubber particle protein (1e^−63^) [*H. brasiliensis*]	F: TTGGAATTTGTACAAGCGACT R: CAAACACCTTGGCAATTCTC	400	~700	63.2
Hb-SNP12-225	0.2967	0.2258	0.2493	Latex cystatin (1e^−53^) [*H. brasiliensis*]	F: GAAAGTGGTGAATGCAAAGC R: AGATGTAACCATTCATAAATATCCA	368	~1,500	60
Hb-SNP12-360	0.2544	0.0417	0.2181					
Hb-SNP12-417	0.3883	0.2727	0.3093					
Hb-SNP12-430	0.2821	0.1515	0.2392					
Hb-SNP12-439	0.3075	0.2273	0.2417					
Hb-SNP12-453	0.4188	0.2581	0.3272					
Hb-SNP13-258	0.4543	0.2647	0.3475	Rubber elongation factor protein (3e^−91^) [*H. brasiliensis*]	F: CATCCATCCATCCGAATTT R: TCAAGGACGCATCTATCCA	466	~470	60
Hb-SNP13-309	0.4611	0.2121	0.351					

The SNP markers developed in this work may be powerful tools for genetic and QTL mapping because they are likely located in sequences that encode proteins related to the stress response and developmental processes in the rubber tree. Some of these SNPs might also be associated with desired traits and could therefore be used as functional markers for marker-assisted selection in *H. brasiliensis* breeding programs.

## Conclusions

The use of EST sequences for the development of molecular markers enables the generation of gene-associated markers, thereby providing a means for the construction of more informative high-density genetic maps. Although cDNA libraries yield a lower number of sequences than NGS technologies, our work shows that these libraries remain a rich source of SSR and SNP markers and can reveal the existence of unknown transcripts. The EST-SSR and SNP markers developed here are a valuable resource for genetic diversity studies, linkage mapping, QTL identification, gene-based association studies, functional analysis of candidate genes and marker-assisted selection in rubber tree genetic studies and breeding programs. These markers are also a powerful tool for evaluating the genetic variability of other *Hevea* species, which are a valuable asset for the genetic improvement of cultivated *H. brasiliensis* clones.

## Electronic supplementary material

Below is the link to the electronic supplementary material.

**Online Resource 1:** Table S1—*H. brasiliensis* genotypes used for the characterization of EST-SSR and SNP markers; Table S2—Quantitative RT-PCR primer sequences (the primers’ sequences and amplicon lengths); Table S3—Summary statistics of the EST sequences generated (statistical description of the processed ESTs, i.e., number of sequenced clones, contigs and singletons; average contig and singleton length; etc.); Table S4—The most highly represented sequences in the cDNA libraries (the contigs that contained 20 or more ESTs); Figure S1—Expression analysis of the most highly represented sequences in the cold-stressed leaf libraries in the clones PB 217, PR 255, GT 1 and IAN 873 A—hypothetical protein (HYPOT), B—ATP synthase CF0 C subunit (CF0), C—NAD(P)H-quinone oxidoreductase subunit H (NADH), D—chloroplast photosystem II 10 kDa polypeptide (PsbR), E and F—indole-3-acetic acid-induced proteins (ARG2-1 and ARG2-2); Fig. S2—BLASTX species distribution for the analyzed sequences; Table S5—The most abundant KEGG pathways represented by the annotated unigenes (i.e., the pathways represented by 15 or more ESTs); Table S6—The frequency of the identified SSR motifs (the frequencies of each SSR motif identified in this study are listed). (PDF 1,177 kb)

**Online Resource 2:** List of the EST-SSR markers characterized in this study. This file contains all of the EST-SSR markers developed here and shows their repeat motifs, primer sequences, primer annealing temperatures, number of alleles, expected and observed heterozygosities, PIC values, transferability to other *Hevea* species and BLASTX hits. (PDF 55 kb)
