# Supplementary material for: Leaf-, panel- and latex-expressed sequenced tags from the rubber tree (Hevea brasiliensis) under cold-stressed and suboptimal growing conditions: the development of gene-targeted functional markers for stress response
Source: Mol Breed. 2014 Apr 29;34(3):1035–53. doi: 10.1007/s11032-014-0095-2 (PMC4162974; doi:10.1007/s11032-014-0095-2)
Supplement: Supplementary file 1 — Online Resource 1: Table S1—H. brasiliensis genotypes used for the characterization of EST-SSR and SNP markers; Table S2—Quantitative RT-PCR primer sequences (the primers’ sequences and amplicon lengths); Table S3—Summary statistics of the EST sequences generated (statistical description of the processed ESTs, i.e., number of sequenced clones, contigs and singletons; average contig and singleton length; etc.); Table S4—The most highly represented sequences in the cDNA libraries (the contigs that contained 20 or more ESTs); Figure S1—Expression analysis of the most highly represented sequences in the cold-stressed leaf libraries in the clones PB 217, PR 255, GT 1 and IAN 873 A—hypothetical protein (HYPOT), B—ATP synthase CF0 C subunit (CF0), C—NAD(P)H-quinone oxidoreductase subunit H (NADH), D—chloroplast photosystem II 10 kDa polypeptide (PsbR), E and F—indole-3-acetic acid-induced proteins (ARG2-1 and ARG2-2); Fig. S2—BLASTX species distribution for the analyzed sequences; Table S5—The most abundant KEGG pathways represented by the annotated unigenes (i.e., the pathways represented by 15 or more ESTs); Table S6—The frequency of the identified SSR motifs (the frequencies of each SSR motif identified in this study are listed). (PDF 1,177 kb) [file 11032_2014_95_MOESM1_ESM.pdf]

Table S1 – *Hevea brasiliensis* genotypes used for the characterizations of EST-SSR and SNP markers

Genotypes[1]/markers	Parental	Origin
**SNPs**		
RRIM 600	Tjir 1 x PB 86	Malaysia
RRIM 606	Tjir 1 x PB 49	Malaysia
RRIM 729	RRIM 623 (PB 49 x Pil B 84) x Fx 25 (F 351 x AVROS 49)	Malaysia
RRIM 805	self pollination of RRIM 628 [Tjir 1 x RRIM 527 (Pil B 50 x Pil B 84)]	Malaysia
RRIM 915	RRIM 605 (Tjir 1 x PB 49) x PB 5/51 (PB 56 x PB 24)	Malaysia
RRIM 937	PB 5/51 (PB 56 x PB 24) x RRIM 703 [RRIM 600(Tjir 1 x PB 86) x RRIM 500 (Pil B 84 x Pil A 44)]	Malaysia
PB 260	PB 5/51 (PB 56 x PB 24) x PB 49	Brazil
PB 346	PB 235 [(PB 5/51(PB 56 x PB 24) x PB S/78] x PB 218	Brazil
Fx 4098	PB 86 x FB 110	Brazil
RO 38	Primary clone	Brazil
CMB 104	IRCA 109 [PB 5/51 (PB 56 x PB 24) x RRIM 600((Tjir 1 x PB 86)] x PFB-5	Brazil
CMB 114	IRCA 109 (PB 5/51 (PB 56 x PB 24) x RRIM 600((Tjir 1 x PB 86)] x PFB-5	Brazil
IAC 307	AVROS 1328 (AVROS 214 x AVROS 317) x PR 107	Brazil
IAC 309	RRIM 626 (Tjir 1 x RRIM 600) x Fx 25 (F 351 x AVROS 49)	Brazil
IAC 500	RRIM 600 (Tjir 1 x PB 86)	Brazil
IRCA 230	GT 1 x PB 5/51 (PB 56 x PB 5/78)	Ivory Coast
IRCA 707	PB 235 [PB 5/51(PB 56 x PB 24) x PB S/78)] x MDF 38	Ivory Coast
IRCA 1159	PB 260 [(PB 5/51 (PB 56 x PB 24) x PB 49] x RO 38	Ivory Coast
**EST-SSRs and SNPs**		
GT 1	Primary clone	Indonesia
PB 235	PB 5/51 (PB 56 x PB 24) x PB S/78	Brazil
PB 217	PB 5/51 (PB 56 x PB 24) (PB 56 x PB 24) x PB 69	Brazil
PB 233	PB S/15 x PB 5/63 (PB 56 x PB 24)	Brazil
PB 311	RRIM 600 (Tjir 1 x PB 86) x PB 235 [PB 5/51 (PB 56 x PB 24) x PB S/78]	Brazil
PC 140	PB 5/51 (PB 56 x PB 24) x RRIM 703 [RRIM 600 (Tjir 1 x PB 24) x RRIM 500 (Pil B 84 x Pil A 44)	Malaysia
PR 255	Tjir 1 x PR 107	Indonesia
RRIM 701	44/553 x RRIM 501(Pil A 44 x Lun N)	Malaysia
RRIM 809	RRIM 600 (Tjir 1 x PB 86) x RRIM 623 (PB 49 x Pil B 84)	Malaysia
RRIM 728	GT 1 x RRIM 623((PB 49 x Pil B 84)	Malaysia
RRIM 913	PB 5/51 (PB 56 x PB 24) x RRIM 623 (PB 49 x Pil B 84)	Malaysia
RRII 105	Tjir 1 x Gl1	India
RRIC 100	RRIC 52 x PB 86	Sri Lanka
IAC 306	AVROS 49 x RRIM 509 (Pil A 44 x Lun N)	Brazil
IAC 313	RRIM 626 [Tjir 1 x RRIM 600(Tjir 1 x PB 86)] x Fx 25 (F 351 x AVROS 49)	Brazil
IAC 318	RRIM 600 (Tjir 1 x PB 86) x Fx 3899 (F 4542 x AVROS 363)	Brazil

IRCA 27	F 4542 x AVROS 363	Ivory Coast
IRCA 209	GT 1 x RRIM 605 (Tjir 1 x PB 49)	Ivory Coast
**EST-SSRs**		
Hevea guianensis	Wild species	Brazil
Hevea. rigidifolia	Wild species	Brazil
Hevea nítida	Wild species	Brazil
Hevea pauciflora	Wild species	Brazil
Hevea benthamiana	Wild species	Brazil
Hevea camargoana	Wild species	Brazil

(1)IAC – Instituto Agronômico de Campinas; IAN: Instituto Agronômico do Norte; F: Ford (clone primário); Fx: Ford cross; AVROS: Algemene Veriniging Rubberplanters Oostkust Sumatra; Tjir: Tjirandji; RO: Rondônia; RRIM: Rubber Research Institute of Malaysia; Pil: Pilmor; Lun: Lunderston; PB: Prang Besar; GT: Godang Tapen; PR: Proesfstation voor Rubber; RRII: Rubber Research Institute of India; RRIC: Rubber Research Institute of Ceylon; FB: Ford Belém; F: Ford; Gl: Glenshield; IRCA: Institut des Recherches sur e Caoutchouc; PC: Promotion Clone; CMB - Cirad Michelin Brasil; PFB: Pé Franco de Belterra.

Table S2 – Quantitative RT-PCR primer sequences.

Primer	Primer Sequence (5' – 3')	Amplicon Length (bp)
CF0	F - ATTGATTTCTGCCGCTTCC<br>R - GGGTTGTCTTGCGATACCTTC	121
HYPOT	F - TGGAAGGCTAGGGGTTATAGTC<br>R - TTCCATAAAGGAGCCGAATG	101
PsbR	F - ATATGGAGCCAACGTGGATG<br>R - CACTGCCCATATTGCCAAAC	109
NADH	F - GTTCTTTACTTGGGCGGTTG<br>R - CAATTGTTGTTCCAAAGACTCC	100
ARG2-1	F – AACTGCGGAGGAGAAGATTG<br>R – TCCTCCGCTAAATTCTCTGG	93
ARG2-2	F - TGGCTCGCTCTTTCTCAAAC<br>R - AGGCTGGACACAACTCCTTG	112
GAPDH	F - AGTGCACCGATGTTTCTTCC<br>R - CTTGCTGGCTACCAAATGAG	143

CF0 - ATP synthase CF0 C subunit, HYPOT - hypothetical protein, PsbR - chloroplast photosystem II 10 kDa polypeptide, NADH - NAD(P)H-quinone oxidoreductase subunit H, ARG2-1 e 2 - indole-3-acetic acid-induced proteins.

Table S3 – Summary statistics of the EST sequences generated

	Numbers
Total number of clones sequenced	10,080
Number of high-quality EST sequences	8,263 (82%)
Redundancy of ESTs	41%*
Contigs	816
Number of ESTs in contigs (range)	2 – 162
Total number of ESTs in contigs	4,054 (49.1%)
Average number of ESTs per contig	4.97
Average contig length (bp)	826

Singletons	4,209 (50.9%)
Singleton average length (bp)	696
Total unigene sequences	5,025
Redundancy of unigenes	1.5%*

* "No-hit" sequences were excluded from the analysis.

Table S4 – The most highly represented sequences in the cDNA libraries.

Contig	N° of ESTs	Length (bp)	Sequence description (BlastX)	E-value	% identity
98	162	803	hypothetical protein M569_00588, partial [*Genlisea aurea*]	$2e^{-14}$	82
130	88	913	Rubber elongation factor protein [*H. brasiliensis*] (sp|P15252.2|REF_HEVBR)	$6e^{-91}$	100
66	78	2364	Photosystem I assembly protein Ycf4 (*H. brasiliensis*); Photosystem I subunit VIII (*H. brasiliensis*)	$1e^{-93}$; $3e^{-13}$	99; 100
127	74	665	Chloroplast photosystem II 10 KDa polypeptide (*Jatropha curcas*)	$2e^{-65}$	82
101	58	883	Ribulose-1,5-bisphosphate carboxylase small subunit (*H. brasiliensis*)	$4e^{-122}$	99
52	54	1040	Pro-hevein [*H. brasiliensis*] (P02877.2|HEVE_HEVBR)	$2e^{-144}$	99
348	50	2646	ATP Synthase CF0 A subunit [*H. brasiliensis*] (ADO33548.1)<br>ATP synthase CF0 C subunit [Nicotiana tabacum] (NP_054483.1)	$6e^{-127}$<br>$4e^{-28}$	99<br>100
81	47	1094	Indole-3-acetic acid induced protein ARG2, putative [*Ricinus communis*] (EEF39286.1)	$7e^{-31}$	70
364	47	2344	Photosystem II protein K (*Camellia sinensis*); Photosystem II protein I (*Magnolia kwangsiensis*)	$3e^{-20}$; $3e^{-18}$	91; 88
366	47	1194	*Manihot esculenta* EST (DB938126.1)	0	92 (BlastN)

152	46	2585	NAD(P)H-quinone oxidoreductase subunit H [*Medicago truncatula*] (AES86356.1)	$7e^{-148}$	90
246	44	2724	ATP-dependent Clp protease proteolytic subunit [*H. brasiliensis*] (ADO33581.1)	$3e^{-57}$	97
126	39	655	Metallothionein [*Hevea brasiliensis*] (ACT68013.1)	$9e^{-27}$	98
284	37	1190	ATP Synthase CF0 C subunit [*Nicotiana tabacum*] (NP_054483.1)	$3e^{-28}$	100
60	36	1028	Small rubber particle protein [*H. brasiliensis*] (sp|O82803.1|SRPP_HEVBR)	$2e^{-138}$	100
491	35	606	Probable non-specific lipid-transfer protein AKCS9-like [*Glycine Max*] (XP_003555069.1)	$6e^{-25}$	61
177	34	1953	Ribossomal protein L16 [*Ximenia americana*] (ADD30329.1)	$9e^{-38}$	67
144	34	1904	NADH dehydrogenase subunit I [*Manihot esculenta*] (YP_001718490.1);	$3e^{-111}$	99
			NAD(P)H-quinone oxidoreductase subunit [*Medicago truncatula*] (XP_003621687.1)	$4e^{-57}$	62
185	33	2126	Ribossomal protein L33 (*H. brasiliensis*); Photosystem I subunit IX (*Populus trichocarpa*)	$2e^{-35}$; $1e^{-14}$	100; 100
			Ribosomal protein S18 [*Ricinus communis*] (AEJ82577.1)	$3e^{-39}$	97
288	31	435	metallothionein 3-like protein [*Hevea brasiliensis*] (ADR30789.1)	$1e^{-24}$	91
71	30	850	REF-like stress related protein 2 [*H. brasiliensis*] (gb|AAP46160.1)	$1e^{-61}$	99
42	26	496	*H. brasiliensis* EST (JG294355.1)	0	99 (BlastN)
89	25	386	*Hevea brasiliensis* latex abundant protein 1 (LAP1) mRNA, complete cds (gb|AY221986.1)	0	100 (BlastN)
20	24	1211	NADH dehydrogenase subunit 4 (*H. brasiliensis*)	0	99
27	24	690	Hypothetical protein (*Medicago truncatula*)	$1e^{-16}$	91
493	23	476	Conserved hypothetical protein [*Ricinus communis*] (ref|XP_002513861.1)	$8e^{-09}$	59

142	20	598	*H. brasiliensis* EST (gb|JG005167.1)	0	99 (BlastN)

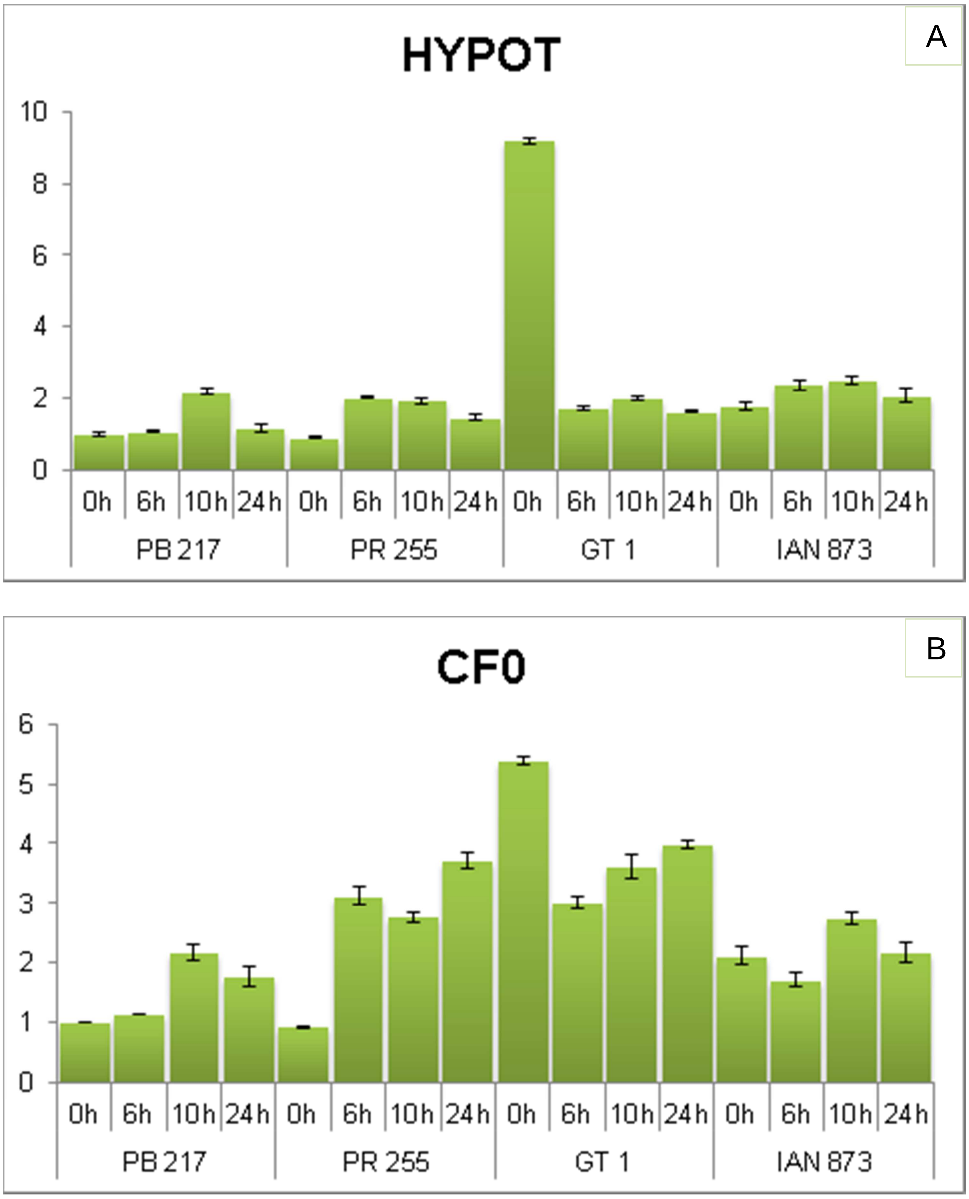

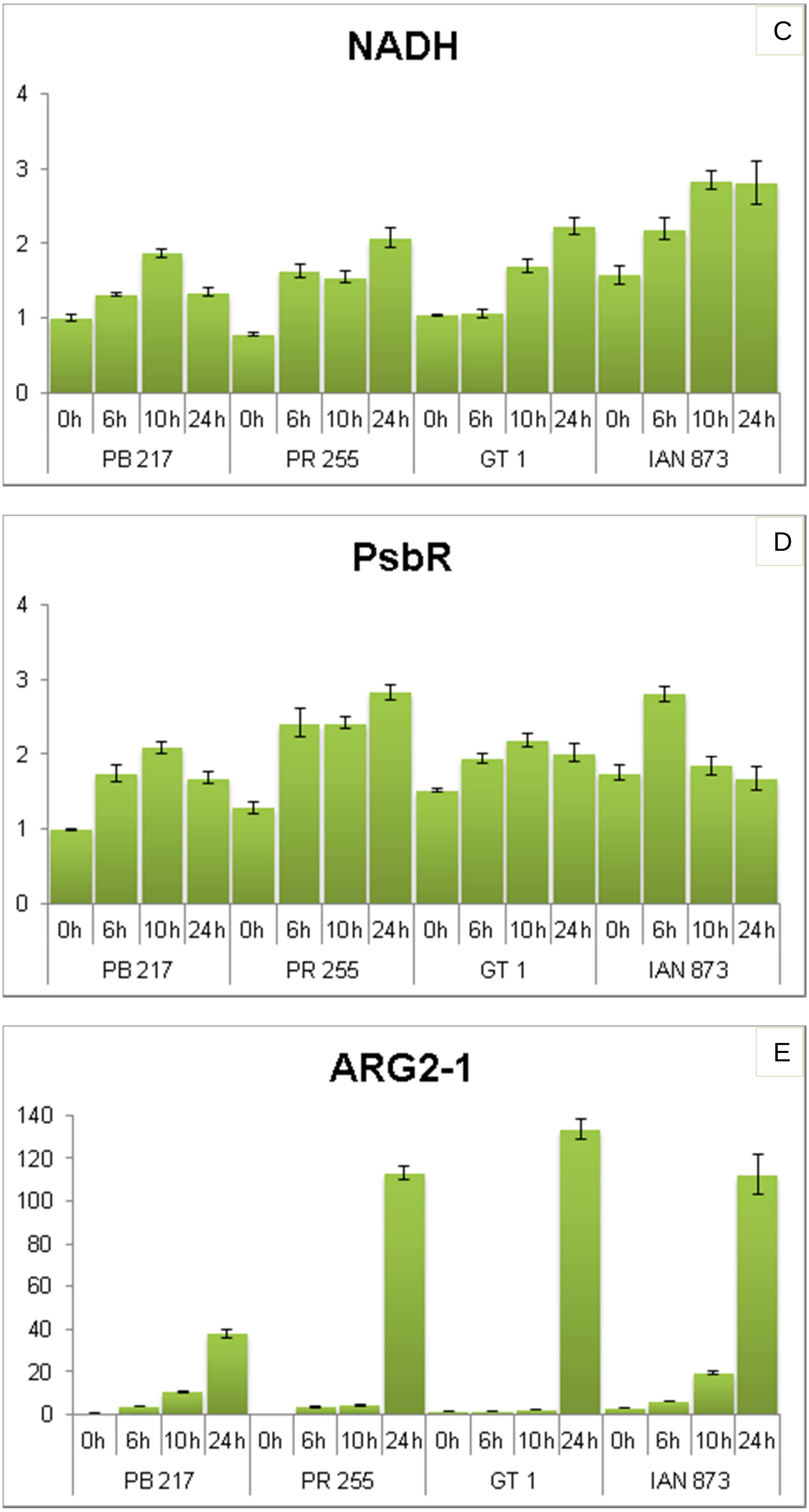

C
NADH
D
PsbR
E
ARG2-1
0h
6h
10h
24h
PB 217
PR 255
GT 1
IAN 873

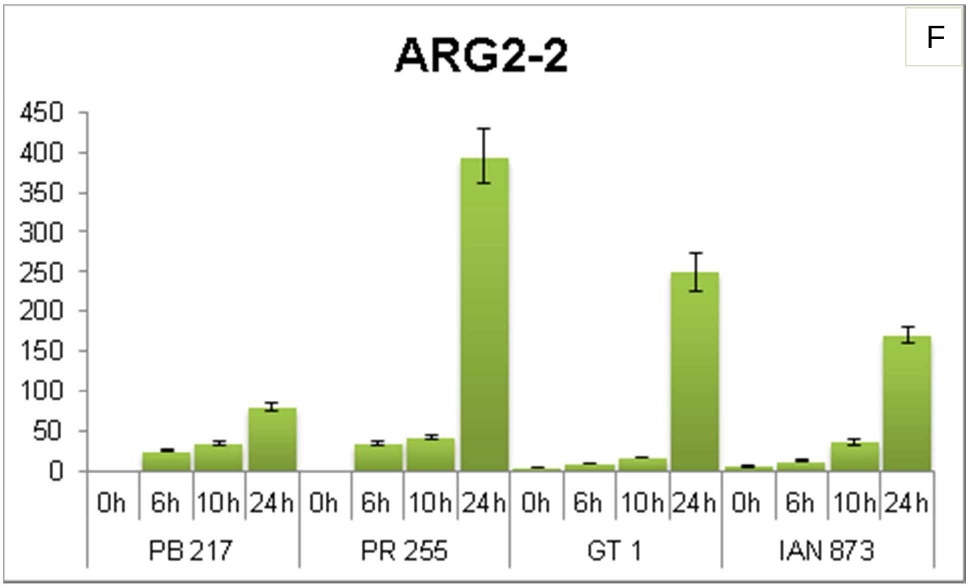

Figure S1 – Expression analysis of the most highly represented sequences in the cold-stressed leaves libraries in the clones PB 217, PR 255, GT 1 and IAN 873. A – hypothetical protein (HYPOT), B - ATP synthase CF0 C subunit (CF0), C - NAD(P)H-quinone oxidoreductase subunit H (NADH), D - chloroplast photosystem II 10 kDa polypeptide (PsbR), E and F - indole-3-acetic acid-induced proteins (ARG2-1 and ARG2-2).

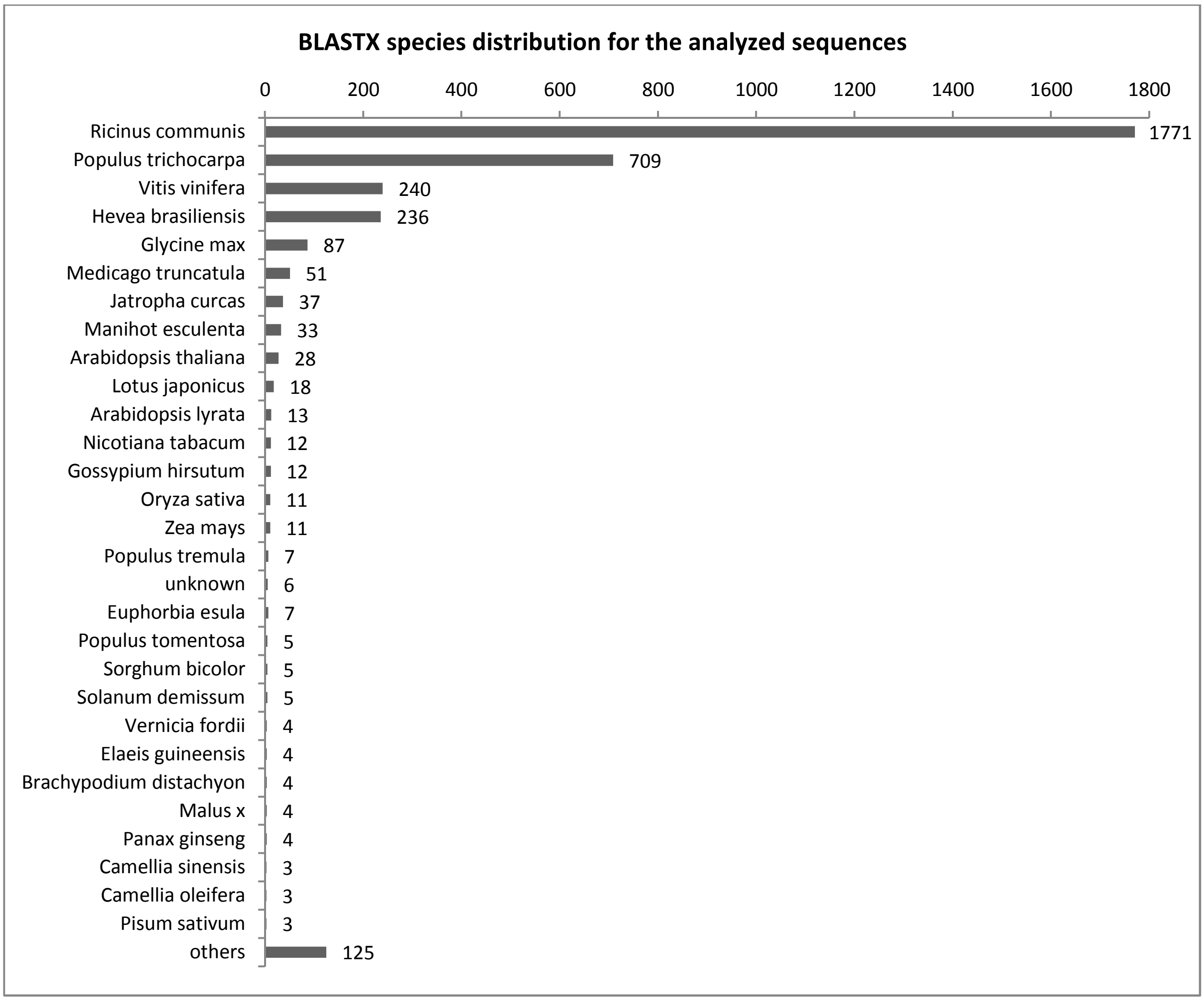

Figure S2 – BLASTX species distribution for the analyzed sequences

Table S5 – The most abundant KEGG pathways represented by the annotated unigenes

	Pathways	N° of sequences	N° of enzymes
1	Carbohydrate metabolism	158	81
2	Purine metabolism	51	18
3	Carbon fixation in photosynthetic organisms	45	18
4	Nitrogen metabolism	40	12
5	Oxidative phosphorylation	34	7
6	Phenylpropanoid biosynthesis	29	4
7	Pyrimidine metabolism	27	11
8	Phenylalanine metabolism	27	10
9	Flavonoid biosynthesis	26	13
10	Cysteine and methionine metabolism	22	10
11	Glycerolipid metabolism	20	12
12	Glycrophospholipid metabolism	19	12
13	Arginine and proline metabolism	18	10
14	Glutathione metabolism	15	7
15	Glycine, serine and threonine metabolism	15	8
16	Thiamine metabolism	15	5

Table S6 – The frequency of the identified SSR motifs.

Motif	N° of SSRs	Total (%)
AG/CT	167	28.4
AT/TA	107	18.2
AAG/CTT	82	13.9
AAT/ATT	50	8.5
AC/TG	27	4.6
ATC/GAT	24	4.1
AAAT/ATTT	23	3.9
AGC/GCT	18	3.1
AGG/CCT	11	1.9

AAAAT/ATTTT	11	1.9
ACC/GGT	8	1.4
AAAAG/CTTTT	7	1.2
AAAG/CTTT	5	0.9
AATT/TTAA	5	0.9
AAC/TTG	5	0.9
AAAC/GTTT	4	0.7
AAAAAT/ATTTTT	4	0.7
CCG/CGG	3	0.5
AACTC/GAGTT	3	0.5
AAATT/AATTT	3	0.5
ACG/CGT	2	0.3
ACT/AGT	2	0.3
AATCGG/CCGATT	2	0.3
CG/GC	1	0.2
AACC/GGTT	1	0.2
ATAG/CTAT	1	0.2
ATCTC/GAGAT	1	0.2
AAAAC/GTTTT	1	0.2
AAAGG/CCTTT	1	0.2
AATCAC/GTGATT	1	0.2
AGAGGC/GCCTCT	1	0.2
AAAAAG/CTTTTT	1	0.2
AAAAGG/CCTTTT	1	0.2

AATGGC/GCCATT	1	0.2
ACCTCC/GGAGGT	1	0.2
AATTCC/GGAATT	1	0.2
AGCTCC/GGAGCT	1	0.2
AATTAT/ATAATT	1	0.2
