# Supplementary material for: Leaf-, panel- and latex-expressed sequenced tags from the rubber tree (Hevea brasiliensis) under cold-stressed and suboptimal growing conditions: the development of gene-targeted functional markers for stress response
Source: Mol Breed. 2014 Apr 29;34(3):1035–53. doi: 10.1007/s11032-014-0095-2 (PMC4162974; doi:10.1007/s11032-014-0095-2)
Supplement: Supplementary file 2 — Online Resource 2: List of the EST-SSR markers characterized in this study. This file contains all of the EST-SSR markers developed here and shows their repeat motifs, primer sequences, primer annealing temperatures, number of alleles, expected and observed heterozygosities, PIC values, transferability to other Hevea species and BLASTX hits. (PDF 55 kb) [file 11032_2014_95_MOESM2_ESM.pdf]

| Primer | Motif | Primer sequences (5' - 3') | Ta (°C) | Expected length (bp) | Observed length (range, bp) | N° of alleles | He | Ho | PIC | Cross-species amplification | Analysis method | BLASTX hit |
|---|---|---|---|---|---|---|---|---|---|---|---|---|
| EHBp-1 | $(CA)_7$ | F - GCATCCTCAATCTCTCAAAG<br>R - AGTGTAGCAGCTGTAGCAAGT | 60 | 210 | 208-215 | 4 | 0.6578 | 0.4 | 0.607 | all | Acrilamide | no hit |
| EHBp-2 | $(TAAT)_4$ | F - CACGACGTTGTAAAACGACCATAAAAATGAATCACACTCACG<br>R - ACCACCATCCTCCACAACTC | TD1 | 209 | 209-220 | 3 | 0.3711 | 0.4667 | 0.3227 | all | Fluorescence | Cucumber peeling cupredoxin |
| EHBp-3 | $(CT)_6$ | F - CACGACGTTGTAAAACGACCTTGTTTATTTGCCGCTGCT<br>R - TGTTCTGCCAACCCACTGTA | TD1 | 242 | 250-255 | 2 | 0.4911 | 0.3333 | 0.3705 | H. gui | Fluorescence | S-adenosylmethionine synthase 2 |
| EHBp-4 | $(GAT)_7$ | F - GGTTATGGGTGTTTTGCAAGT<br>R - CCAACTACACCCACCAACAA | 63 | 236 | 236-239 | 2 | 0.51 | 0.5 | 0.3737 | all | Acrilamide | no hit |
| EHBp-5 | $(CT)_8$ | F - GGGTGTAGATGAGGACAGCAA<br>R - GCAACCGTGCAAGGATTTAT | 63 | 225 | 219-231 | 3 | 0.63 | 0.67 | 0.541 | H. gui, H. nit, H. rig, H. pau, H. bent | Acrilamide | no hit |
| EHBp-6 | $(AG)_{14}$ | F - GATACCCACCCCAAGAACAA<br>R - TGCCAAAGCTGATGGTTTTA | 63 | 225 | 209-229 | 7 | 0.83 | 0.76 | 0.7852 | all | Acrilamide | hypothetical protein |
| EHBp-7 | $(TTC)_5$ | F - CAGGTCCAGATCTGCTGACA<br>R - CAAAAAGGATCGGAATTGGA | 63 | 228 | 230-233 | 2 | 0.06 | 0.06 | 0.0526 | all | Acrilamide | no hit |
| EHBp-8 | $(GAA)_7$ | F - CCACCAGGTGCAACTTGATA<br>R - CCATTGCTTCAGACATAGCA | 63 | 248 | 245-257 | 4 | 0.55 | 0.67 | 0.4438 | all | Acrilamide | predicted protein (LIGHT SENSITIVE HYPOCOTYLS 3) |
| EHBp-9 | $(AAAG)_6$ | F - GTCTCAAGCTCAATAGGAGAAT<br>R - GCGCAAATCATGAAAGAAG | 63 | 170 | 170-176 | 3 | 0.38 | 0.22 | 0.3402 | all | Acrilamide | rapid alkalinization factor 1 |
| EHBp-10 | $(AATT)_5$ | F - GCATCAAGTGCCACGAGTT<br>R - CAAAAGGCCAAGGCACTAAT | 63 | 231 | 231-239 | 3 | 0.16 | 0.06 | 0.1494 | H. gui, H. rig, H. nit, H. bent, H. cam | Acrilamide | no hit |
| EHBp-11 | $(TAAAAA)_4$ | F - TGATGCAACGTTAACACACG<br>R - TGGAAGATAAAGGGCAAATCA | 63 | 238 | 238 | 1 | 0 | 0 | 0 | H. gui, H. rig, H. nit, H. bent | Acrilamide | conserved hypothetical protein |
| EHBp-12 | $(CAC)_6$ | F - TCACTGCCTTCCAATTCTCC<br>R - CATCCTTGCCCTTTTTCAAG | 62 | 206 | 200-209 | 2 | 0.36 | 0.22 | 0.2859 | all | Acrilamide | probable plastid-lipid-associated protein 4, chloroplastic |
| EHBp-13 | $(TTTA)_4$ | F - CGGAACTACTTCCAAAACTTGA<br>R - GAATTCCCAATAGCGTTTCG | 63 | 240 | 240-244 | 2 | 0.29 | 0.22 | 0.2392 | all | Acrilamide | Lipoxygenase 1 |
| EHBp-14 | $(ACT)_5$ | F - CCTTCCATCTCCAGCATAACA<br>R - CTTCGATTCCCCGAGATACA | 63 | 247 | 247-253 | 3 | 0.64 | 0 | 0.5443 | all | Acrilamide | no hit |
| EHBp-15 | $(AG)_{13}$ | F - TGCAAGTGAGAAAGCAAAGAA<br>R - GAAACGGCGAAATATTGCAT | 57 | 201 | 199-213 | 5 | 0.7 | 0.89 | 0.6352 | all | Acrilamide | acyl-carrier-protein |
| EHBp-16a | $(TC)_{12}$ | F - GCGAAAATTGGTTTTTGGTT<br>R - TCAAGCTACACACAGAGAGAAA | 55 | 204 | 206-228 | 4 | 0.41 | 0.33 | 0.3538 | H. gui, H. nit, H. pau, H. bent, H. cam | Acrilamide | no hit |
| EHBp-16b | $(TAT)_5$ | F - AAAAAGGAAGCTTTTGCTTAGT<br>R - CAAGGAAACAGTTCATGCAAA | 63 | 215 | 215 | 1 | 0 | 0 | 0 | all | Acrilamide | |

| | | | | | | | | | | | | |
|---|---|---|---|---|---|---|---|---|---|---|---|---|
| EHBp-17 | (TCT)$_5$ | F - CCTCCTCAAAACCCCTCAA<br>R - ATGGAAAGGGAGCGAAAGAT | 63 | 202 | 204-216 | 4 | 0.68 | 0.76 | 0.5972 | all | Acrilamide | 60S ribosomal protein L34 |
| EHBp-18 | (TC)$_{13}$ | F - GGACCAAACGCTCACTGTCT<br>R - GGGAGAAAATGCAGATCCAA | 63 | 212 | 204-224 | 5 | 0.73 | 1 | 0.66 | all | Acrilamide | vesicle-associated membrane protein |
| EHBp-19 | (ATA)$_6$ | F - TGTGCAACACATACAAGCTGA<br>R - TTGGACATTTGGCGTTGTTA | 63 | 179 | 179 | 1 | 0 | 0 | 0 | all | Acrilamide | no hit |
| EHBp-20 | (GCT)$_5$ | F - CACGACGTTGTAAAACGACGGACAGAGCAAAGTGGGATG<br>R - TTTTGAACACAAGGAACCTACAG | TD3 | 169 | 300-303 | 2 | 0.0556 | 0.0556 | 0.0526 | all | Fluorescence | acyl-CoA-binding protein |
| EHBp-21 | (TC)$_7$ | F - CACGACGTTGTAAAACGACTTCATCTTCTCAGGCCAAAA<br>R - ACTGAAACCCACCTCACCAA | TD2 | 207 | 207-223 | 6 | 0.7077 | 0.2353 | 0.6419 | all | Fluorescence | Jasmonate-zim-domain protein 3, putative isoform 1 |
| EHBp-22 | (TAT)$_6$ | F - CACGACGTTGTAAAACGACGCAATTAAGGATGGCAAGGA<br>R - CAATTGAAATCAAATGAACCA | TD2 | 171 | 171-174 | 2 | 0.4365 | 0.3889 | 0.3343 | H. gui, H. nit, H. pau, H. bent, H. cam | Fluorescence | pathogenesis-related protein STH-2 |
| EHBp-23 | (TTTG)$_4$ | F - CACGACGTTGTAAAACGACTTTCATTGGAGCACTTGCAG<br>R - AAAGAGTCATCATCATAATCCTCTCA | TD2 | 267 | 263-267<br>229-239 | 3<br>4 | 0.5952<br>0.6175 | 0.4444<br>0.6111 | 0.5056<br>0.5576 | all | Fluorescence | aquaporin |
| EHBp-24 | (AAG)$_5$ | F - CACGACGTTGTAAAACGACATTCCTGCAAGAGGCTGAGA<br>R- TCCAGGTTTTTCTTCGTGG | TD2 | 210 | 207-210 | 2 | 0.0556 | 0.0556 | 0.0526 | all | Fluorescence | AUX/IAA transcriptional regulator family protein isoform 1 |
| EHBp-25 | (CAG)$_5$ | F - CACGACGTTGTAAAACGACTCCAAGTTCCATTCCCAAAC<br>R - TAACGGTGTCAGCCAATGAA | TD2 | 253 | 248-263 | 3 | 0.5952 | 0.0556 | 0.496 | all | Fluorescence | WRKY transcription factor |
| EHBp-26 | (ATT)$_5$ | F - CACGACGTTGTAAAACGACCCCTCCTATGTTTGCAGAAG<br>R - TCGGAACAGATATATTCTCACAATTC | TD2 | 257 | 257 | 1 | 0 | 0 | 0 | all | Fluorescence | eukaryotic translation initiation factor 2 |
| EHBp-27 | (AAG)$_7$ | F - TCTTCAAAGCTTCAACAGCAAC<br>R - CTTCGCTCCAAAAACTCCAT | 63 | 249 | 240 - 286 | 10 | n/a | n/a | n/a | all | Acrilamide | probable beta-D-xylosidase 5-like |
| EHBla-1 | (ATT)$_5$ | F - CTTTCAGCCAAGTTGCATCA<br>R - ATCTCGTCGGCATCAACTTC | 63 | 172 | 172-175 | 2 | 0.11 | 0 | 0.0994 | all | Acrilamide | NADH dehydrogenase [ubiquinone] 1 alpha subcomplex subunit 1 |
| EHBla-2 | (AAT)$_{17}$ | F - CGACTGCTGCTGTGACAAAT<br>R - CAGGAACACAACGTCTTCTTTT | 63 | 212 | 182-218 | 8 | 0.73 | 0.88 | 0.6734 | H. gui, H. nit, H. pau, H. bent | Acrilamide | conserved hypothetical protein |
| EHBla-3 | (GAT)$_5$ | F - TGGTTAATCCCATGATGCAA<br>R - CCTGAGCCATATGGATGGAC | 60 | 220 | 220 | 1 | 0 | 0 | 0 | all | Acrilamide | putative clathrin assembly protein At5g35200 isoform 1 |
| EHBla-4 | (GA)$_7$ | F - CGGCAAATCATTTTGGTCTT<br>R - AACTGGTGGAGGATTTGCAC | 63 | 245 | 386-392 | 2 | 0.51 | 0 | 0.3719 | all | Capillary Electrophoresis | ENTH/VHS/GAT family protein isoform 1 |
| EHBla-5a | (CT)$_8$ | F - CACGACGTTGTAAAACGACGCAAAGCCTTAAACGCAAAG<br>R - CCTTCTCTCTTCTTCTGGGCTA | TD2 | 123 | 123-135 | 3 | 0.1603 | 0.0556 | 0.1494 | all | Fluorescence | NC domain-containing protein-related |
| EHBla-5b | (GAA)$_{12}$ | F - CACGACGTTGTAAAACGACGCCCAGAAGAAGAGAGAAGGA<br>R - CCCTGGCTTCAACTCATCTC | TD2 | 127 | 115-136 | 6 | 0.7094 | 0.8235 | 0.636 | all | Fluorescence | |
| EHBla-6 | (AAG)$_{10}$ | F - CACGACGTTGTAAAACGACTTGATGGCCAATTATCAGCA<br>R - CTTTCTTCGTCGTTGCCTTC | TD2 | 223 | 214-238 | 6 | 0.7016 | 0.7778 | 0.6283 | H. gui, H. rig, H. nit, H. cam | Fluorescence | conserved hypothetical protein |
| EHBla-7 | (TC)$_{12}$ | F - CACGACGTTGTAAAACGACGCAAAGACAAATTAACaATCAACG | TD2 | 166 | 154-166 | 4 | 0.7206 | 0.8889 | 0.6457 | all | Fluorescence | membrane-anchored ubiquitin-fold |

| | | | | | | | | | | | | |
|---|---|---|---|---|---|---|---|---|---|---|---|---|
| | | R - AAAGGAAAATCGCTGGATCA | | | | | | | | | | protein 3 |
| EHBla-8 | $(AT)_8$ | F - CACGACGTTGTAAAACGACGGATTGGGGGAGAGAAAACT<br>R - CAAAGCCTTCATCTTCCGACT | TD2 | 177 | 174-180 | 2 | 0.4365 | 0.1667 | 0.3343 | H. gui, H. rig, H. nit, H. pau, H. bent | Fluorescence | conserved hypothetical protein |
| EHBla-9 | $(AG)_{10}C(GA)_{10}$ | F - CACGACGTTGTAAAACGACTGGGGCATGAAAAAGCTATC<br>R - AAGGTATTGCGTGCCATTTC | TD2 | 151 | 131-157 | 5 | 0.6349 | 0.6667 | 0.5572 | all | Fluorescence | conserved hypothetical protein |
| EHBla-10 | $(CTT)_6$ | F - CACGACGTTGTAAAACGACGCCATGGCAGCAACATACTA<br>R - TGACCACACAGGTGGAAACT | TD2 | 196 | 190-217 | 4 | 0.554 | 0.2778 | 0.4438 | all | Fluorescence | 2-methyl-6-geranylgeranylbenzoquinone methyltranferase |
| EHBla-11 | $(AT)_8$ | F - CACGACGTTGTAAAACGACGGACAGATCTGGAGGATTCG<br>R - AACCAAGTTTTAAAGCAATTG | TD2 | 213 | 217-229 | 5 | 0.4952 | 0.3333 | 0.446 | H. gui, H. rig, H. nit, H. pau | Fluorescence | CSL zinc finger domain-containing protein |
| EHBla-12 | $(AATT)_4$ | F - CACGACGTTGTAAAACGACTGTGACAAATCTTTTCAAATTTCACT<br>R - TCTTGTCGGAAACTGCCTTT | TD2 | 245 | 325 | 1 | 0 | 0 | 0 | H. gui, H. rig, H. nit, H. pau, H. bent | Fluorescence | EG45-like domain containing protein-like |
| EHBla-13 | $(AG)_7$ | F - CACGACGTTGTAAAACGACAGCAAAAAGAATTTTCAGTCTCT<br>R - TTCTTTGACGCTGGACCTCT | TD2 | 204 | 202-204 | 2 | 0.5127 | 0.3889 | 0.3742 | all | Fluorescence | VAMP-like protein YKT61-like |
| EHBla-14 | $(AG)_{12}$ | F - CACGACGTTGTAAAACGACCAACTCACCAGTTGCTGTCG<br>R - TGAATCAAAACCAGGAGGTG | TD2 | 167 | 157-167 | 3 | 0.2079 | 0.2222 | 0.19 | H. gui, H. nit, H. pau, H. bent, H. cam | Fluorescence | balbiani ring 1-related family protein |
| EHBla-15 | $(GA)_{13}$ | F - CACGACGTTGTAAAACGACGCTGCTTGAACACGAAACTG<br>R - ACAGCGGATTCTCTTGGAAA | TD3 | 227 | 227-239 | 4 | 0.5444 | 0.6667 | 0.456 | all | Fluorescence | conserved hypothetical protein |
| EHBla-16 | $(AGC)_6$ | F - CACGACGTTGTAAAACGACCAGGGGCAGGAAATCATCTA<br>R - CTGAAATGCGCCTTCTCTTC | TD2 | 282 | 273-282 | 3 | 0.6365 | 0.3889 | 0.5462 | all | Fluorescence | ATP binding protein, putative |
| EHBla-17 | $(TC)_8$ | F - CACGACGTTGTAAAACGACGCCGCTACAAGCAATAGAGC<br>R - CATGATTGAGCGTTGGATTG | TD2 | 157 | 157-163 | 3 | 0.5095 | 0.3333 | 0.3972 | all | Fluorescence | Signal recognition particle 14 kDa protein |
| EHBla-18 | $(GT)_7$ | F - CACGACGTTGTAAAACGACCCGTCAACAGCTTCTTCACA<br>R - AACCCATTTCCACCTTTATTTT | TD3 | 223 | 223-227 | 3 | 0.2656 | 0.1765 | 0.2364 | all | Fluorescence | B12D protein |
| EHBla-19 | $(TA)_6$ | F - CACGACGTTGTAAAACGACAATGAAGCACGGGGAAGTTA<br>R - ATGTTGCCAGGGACAATACA | TD2 | 179 | 179-181 | 2 | 0.0556 | 0.0556 | 0.0526 | all | Fluorescence | mannan endo-1,4-beta-mannosidase 7 |
| EHBla-20 | $(GCTGGA)_4$ | F - CACGACGTTGTAAAACGACTGCTTGTCTTGATGCCTTTG<br>R - GGAAGAGCCACCAAAACTC | TD2 | 151 | 139-157 | 4 | 0.6254 | 0.5 | 0.5411 | all | Fluorescence | Werner-syndrome like protein |
| EHBc-1a | $(CT)_6$ | F - CACGACGTTGTAAAACGACGAAGGGACCTGTGGGAAAAC<br>R - GCAAGAGCAAGGATCGCTAA | TD1 | 245 | 239-245 | 2 | 0.5 | 1 | 0.375 | all | Fluorescence | chlorophyll a-b binding protein CP26, chloroplastic-like |
| EHBc-1b | $(AATT)_4$ | F - CACGACGTTGTAAAACGACTCACCTATCCATCTCCTTTCTCA R - CCACTAAACAAAATCCCCTAACC | TD1 | 206 | 206 | 1 | 0 | 0 | 0 | all | Fluorescence | |
| EHBc-2 | $(GAAA)_4$ | F - CACGACGTTGTAAAACGACCACAAACAAAAGAATAACCAATCTG<br>R - TTATTGGCTCGAGGCTCTTG | TD1 | 264 | 264 | 1 | 0 | 0 | 0 | all | Fluorescence | bifunctional nuclease in basal defense response 1 |
| EHBc-3 | $(AT)_6$ | F - CACGACGTTGTAAAACGACTTGCAAGCAGAATGATGGAG R - TGCAATATAAGCCATACAACATCC | TD1 | 174 | 174 | 1 | 0 | 0 | 0 | H. bent | Fluorescence | NDH dependent flow 6 isoform 1 |
| EHBc-4 | $(GAT)_6$ | F - GCAACTCAAAGAAGGGTCCA<br>R - TGAGGAGCCTTCTGCCTTTA | 63 | 269 | 269-275 | 3 | 0.45 | 0 | 0.3957 | all | Acrilamide | predicted protein |

| | | | | | | | | | | | | |
|---|---|---|---|---|---|---|---|---|---|---|---|---|
| EHBc-5 | $(TCA)_8$ | F - CCGGAGACTTCTTTGCTGAC<br>R - CGAGGAAGAAAGGCAATGTC | 63 | 246 | 146-246 | 6 | 0.71 | 0.72 | 0.6466 | all | Acrilamide | eukaryotic translation initiation factor |
| EHBc-6 | $(TTC)_{10}$ | F - GCTGGGTCGATGAGATCTGT<br>R - CACCAAATAATCACCGTCCA | 62 | 169 | 169-193 | 5 | 0.57 | 0.33 | 0.5015 | all | Acrilamide | no hit |
| EHBc-7 | $(TTC)_6$ | F - GAGCGTGATGAGATGGATCA<br>R - GGGAGGGGATGCATAGAT | 60 | 168 | 168 | 1 | 0 | 0 | 0 | all | Acrilamide | ubiquitin-like modifier-activating enzyme 5-like |
| EHBc-8 | $(TCT)_5$ | F - GAGAGGGTGCTTCTTTGTGC<br>R - CCAAACCCAAAAACCAGAAA | 60 | 187 | 187-208 | 5 | 0.69 | 0.61 | 0.6099 | all | Acrilamide | no hit |
| EHBc-9 | $(GA)_{10}$ | F - TGCCGGACACACTTGTAGTT<br>R - GGGAATGACGTGGACTTGTT | 63 | 221 | 227-233 | 3 | 0.16 | 0.17 | 0.1495 | all | Acrilamide | conserved hypothetical protein |
| EHBc-10 | $(CTT)_5$ | F - AGGGGGACACAAAAACCAG<br>R - TGGACCGCTATGAGAAACAA | 63 | 220 | 205-226 | 3 | 0.51 | 0.44 | 0.3972 | all | Acrilamide | no hit |
| EHBc-11 | $(TTC)_6$ | F - GGAGGTCGTCCATAAGGTAGC<br>R - CCTTCCTCTTCTGCCTTGAA | 63 | 201 | 201-228 | 5 | 0.6 | 0.5 | 0.5296 | all | Acrilamide | CTP synthase 1a |
| EHBc-12 | $(TTG)_6$ | F - TGGATGAGTGCCTTTACTCG<br>R - GACGTACCAACGTGGGATAAA | 63 | 158 | 161-170 | 4 | 0.54 | 0.56 | 0.4561 | all | Acrilamide | no hit |
| EHBc-13 | $(AT)_8$ | F - TGCATGAGTAGGGAATGGTG<br>R - ATTCCGTCAACCAATGATCC | 57 | 240 | 238-242 | 3 | 0.41 | 0.06 | 0.3633 | all | Acrilamide | no hit |
| EHBc-14 | $(AT)_{12}$ | F - AGAGAGGTGGTGAAGCTGGA<br>R - AAACAAGTGCCGGAATTCAT | 63 | 182 | 174-184 | 4 | 0.36 | 0.24 | 0.3165 | all | Acrilamide | CBL-interacting protein kinase 16 |
| EHBc-15 | $(CCTCTG)_4$ | F - CACGACGTTGTAAAACGACCTGCTCACACAAGTGGCA<br>R - TGGGGCTTGCGTACTTTTAC | TD2 | 270 | 264-276 | 3 | 0.4902 | 0.1765 | 0.3872 | all | Fluorescence | negative cofactor 2 transcriptional co-repressor, putative |
| EHBc-16 | $(AAGAA)_5$ | F - CACGACGTTGTAAAACGACTGGACTGGGCCTTAATGTTT<br>R - GGTCCAATGACCCAGAGAAA | TD2 | 218 | 218-228 | 2 | 0.1079 | 0.1111 | 0.0994 | all | Fluorescence | F-box protein PP2-A12 |
| EHBc-17 | $(TCC)_7$ | F - CACGACGTTGTAAAACGACCCCGGCGAATCTATGTCT<br>R - ATAGCATGGCCACAAGGAAG | TD3 | 271 | 271 | 1 | 0 | 0 | 0 | all | Fluorescence | WD repeat domain phosphoinositide-interacting protein |
| EHBc-18 | $(AGA)_5$ | F - CACGACGTTGTAAAACGACTAAGCTCCGCTTGCAGTACA<br>R - AAGAAGGTGGGTTTTCCTCTG | TD2 | 162 | 162 | 1 | 0 | 0 | 0 | all | Fluorescence | isomerase peptidyl-prolyl cis-trans isomerase |
| EHBc-19 | $(GAA)_7$ | F - CACGACGTTGTAAAACGACCAAAAATGGTGGAAGGGAGA<br>R - CAAGTTTGCCATTAGGTATTAAA | TD2 | 140 | 137-140 | 2 | 0.1079 | 0 | 0.0994 | all | Fluorescence | photosystem II stability/assembly factor HCF136, chloroplastic-like |
| EHBc-20 | $(CT)_{17}$ | F - CACGACGTTGTAAAACGACTCCCTTTCTCAAGGTGCTTC<br>R - CCCATGTTCTCTGCATCTGA | TD2 | 230 | 220-238 | 5 | 0.7937 | 0.6111 | 0.7327 | all | Fluorescence | histidine-containing phosphotransfer protein 1 isoform 1 |
| EHBc-21 | $(CT)_9$ | F - CACGACGTTGTAAAACGACTTCATCTTCCTCCTCTCTCCTG<br>R - GCTGAGAAGCTCTCCATCGT | TD2 | 223 | 219-227 | 5 | 0.4286 | 0.3333 | 0.393 | all | Fluorescence | xyloglucan endotransglucosylase/hydrolase protein 9 |
| EHBc-22 | $(TAT)_5$ | F - CACGACGTTGTAAAACGACAAACCACATTCCGACTCCTG<br>R - GGATGCTTACTGACGCACAA | TD2 | 268 | 244-268 | 3 | 0.2656 | 0.2941 | 0.2364 | H. gui, H. rig, H. nit, H. pau | Fluorescence | RING-H2 finger protein ATL48-like |
| EHBc-23 | $(CCAATT)_5$ | F - CACGACGTTGTAAAACGACCCGTTTCTCTCCGTCTTTGT | TD2 | 250 | 254-266 | 3 | 0.4381 | 0.4444 | 0.3709 | all | Fluorescence | conserved hypothetical protein |

| | | | | | | | | | | | | |
|---|---|---|---|---|---|---|---|---|---|---|---|---|
| | | R - TTGGGTAATAGTCCGCTTGG | | | | | | | | | | |
| EHBc-24 | $(GAG)_7$ | F - CACGACGTTGTAAAACGACGAAATCGCCGGTAAGTTTGA<br>R - ATCCATTGGGATTCAAGCAA | TD2 | 200 | 194-200 | 3 | 0.1603 | 0.1667 | 0.1494 | all | Fluorescence | conserved hypothetical protein |
| EHBc-25 | $(GAT)_5$ | F - CACGACGTTGTAAAACGACACTCCTCCAGCTGCTGATTC<br>R - CGTTCTTCTGCAGCCTTCTT | TD2 | 125 | 380 | 1 | 0 | 0 | 0 | all | Fluorescence | Elongation factor 1-delta 2 |
| EHBc-26 | $(TTTTAT)_4$ | F - CACGACGTTGTAAAACGACTCCCCAGGCTCAAATGATTA<br>R - AACTCTCGTTTTCGCACTCAA | TD2 | 236 | 236 | 1 | 0 | 0 | 0 | all | Fluorescence | ABC transporter C family member 2 isoform 2 |
| EHBc-27 | $(AG)_{19}$ | F - CACGACGTTGTAAAACGACTGCCACTTCCAAGAGCTACC<br>R - ACACCCATTTTCCCTTTCCT | TD2 | 180 | 158-192 | 7 | 0.7698 | 0.7222 | 0.7173 | all | Fluorescence | senescence-associated family protein |
| EHBc-28 | $(TC)_6$ | F - CACGACGTTGTAAAACGACGAGTCTTTTTGATTGTTTGTCTGC<br>R - GGCAATTGCATTGATTCTGA | TD1 | 212 | 330 | 1 | 0 | 0 | 0 | all | Fluorescence | GroES-like family protein |
| EHBc-29 | $(AAAT)_4$ | F - CACGACGTTGTAAAACGACGATCAAAGGAAGGTGGCAAT<br>R - AAGCCCAAGTCCCAAACATA | TD1 | 196 | 160; 196 | 2 | 0.42 | 0.3333 | 0.3318 | H. gui, H. rig, H. nit, H. pau, H. bent | Fluorescence | no hit |
| EHBc-30 | $(GGGT)_4$ | F - CACGACGTTGTAAAACGACAAAATCTCCTGCGATGCAAT R - TGCTCTGAACCCACCTTACC | TD1 | 250 | 250 | 1 | 0 | 0 | 0 | all | Fluorescence | conserved hypothetical protein |
| EHBc-31 | $(TTC)_5$ | F - CACGACGTTGTAAAACGACCCTCAGAGGAGGCAATTCAC<br>R - CCCATCGATTTAGCCTCTCA | TD1 | 246 | 246-260 | 3 | 0.1267 | 0.1333 | 0.1228 | all | Fluorescence | patellin-6 |
| EHBc-32 | $(TAT)_6$ | F - TTGGCTACCTACCCAGATGC<br>R - ATGTTCCTTGTGCTCCCAAC | 63 | 225 | 225-258 | 7 | 0.76 | 0.61 | 0.6986 | all | Acrilamide | conserved hypothetical protein |
| EHBc-33 | $(TTA)_9$ | F - TCCTGCAACAGAACAACACC<br>R - ATGAAAACAATGCACCCACT | 63 | 248 | 236-248 | 3 | 0.56 | 0.5 | 0.4813 | all | Acrilamide | no hit |
| EHBc-34 | $(CT)_{10}$ | F- ATTCTGGTGGAAATCGAACG<br>R - AAGGCGAGCAAGAAAACTGT | 63 | 236 | 234-270 | 6 | 0.66 | 0.61 | 0.5758 | all | Acrilamide | metallothionein |
| EHBc-35 | $(CT)_6$ - $(AG)_{12}$ | F- GTGAGAAGTGGGATAACG<br>R - TCGTACAAAATCTTCTCGTG | 57 | 190 | 190-196 | 2 | 0.16 | 0.17 | 0.1411 | all | Acrilamide | R3H domain-containing protein |
| EHBc-36 | $(GAA)_6$ | F - GCCCAAAGAGGAAAATGAGA<br>R - TATGCAACCAATGGGCTTTT | 63 | 198 | 198-207 | 2 | 0.11 | 0.11 | 0.0995 | all | Acrilamide | no hit |
| EHBc-37 | $(AGA)_9$ | F - CAGAAGGGGATTTTGATTGG<br>R - CCGTGGAAGAAAAGAACGAG | 63 | 250 | 250-259 | 3 | 0.43 | 0.33 | 0.3491 | all | Acrilamide | conserved hypothetical protein |
| EHBc-38a | $(ATAA)_4$-$(CT)_6$ | F - GCGAAAATTGGTTTTTGGTT<br>R - TCAAGCTACACACAGAGAGAAA | 58 | 191 | 191-199 | 5 | 0.76 | 0.11 | 0.69 | all | Capillary Electrophoresis | no hit |
| EHBc-38b | $(GTTTT)_4$ | F - AAAAAGGAAGCTTTTGCTTAGT<br>R - CAAGGAAACAGTTCATGCAAA | 58 | 169 | 169-174 | 2 | 0.06 | 0.06 | 0.0526 | all | Capillary Electrophoresis | |
| EHBc-39 | $(AT)_7$ | F - GCATTAAGATAGGTGCGATTAC<br>R - AAGTGAAATTGCCGTGATTG | 60 | 214 | 212-235 | 10 | 0.89 | 0.94 | 0.8512 | all | Capillary Electrophoresis | photosystem II core complex proteins psbY, chloroplast precursor |
| EHBc-40 | $(GGC)_5$ | F - CACCTTGCGGAATTTAGGAT<br>R - CTGCTGCTGCTCATTTTCTG | 62 | 195 | 186-192 | 3 | 0.64 | 0.06 | 0.5455 | H. gui, H. rig, H. pau | Capillary Electrophoresis | serine/threonine-protein kinase HT1-like |

| | | | | | | | | | | | | |
|---|---|---|---|---|---|---|---|---|---|---|---|---|
| EHBc-41 | $(AT)_7$ | F - GCCAAAGCCAAATATTAACCA<br>R - TGATCCGGATGGTAATGGAT | 60 | 274 | 274-286 | 8 | 0.82 | 0.67 | 0.7631 | all | Capillary Electrophoresis | Lactoylglutathione lyase |
| EHBc-42 | $(ACAA)_4$ | F - ATTTGGGCAAAGTGATGAAA<br>R - TGATCCAACTGGAGCAACTATG | 63 | 193 | 189-201 | 4 | 0.62 | 0 | 0.5393 | all | Capillary Electrophoresis | lycopene epsilon cyclase, chloroplastic |
| EHBc-43 | $(GAA)_5$ | F - ACCTTCAAGCGTCGAACAAT<br>R - AAGGATTTCCATCGGACAAA | 63 | 222 | 367-394 | 7 | 0.51 | 0.44 | 0.4797 | H. gui, H. rig, H. pau, H. bent, H. cam | Capillary Electrophoresis | conserved hypothetical protein |
| EHBc-44 | $(AATA)_4$ | F - CACGACGTTGTAAAACGACCTGCATTTCCTTGGGAGAA<br>R - GAGCACGACTAATAACAAATCGAA | TD2 | 193 | 190-193 | 2 | 0.0556 | 0.0556 | 0.0526 | all | Fluorescence | 60S ribosomal protein L34, putative |
| EHBc-45 | $(ATT)_{11}$ | F - CACGACGTTGTAAAACGACATGCCACCACCCTTCAAGTA<br>R - TGAGGGGACAAAAATTCCAA | TD3 | 289 | 280-310 | 8 | 0.7841 | 0.7778 | 0.7292 | all | Fluorescence | predicted protein |
| EHBc-46 | $(TG)_9$ | F - CACGACGTTGTAAAACGACTGCTAATGATGCTGGTGGAC<br>R - CATCATGCGTGCCATAAAAT | TD2 | 225 | 211-229 | 5 | 0.5921 | 0.5556 | 0.5031 | H. gui, H. rig, H. nit, H. pau, H. bent | Fluorescence | guanosine-3',5'-bis(diphosphate) 3'-pyrophosphohydrolase, putative |
| EHBc-47 | $(TGC)_5$ | F - CACGACGTTGTAAAACGACATTTTGTGGGATGCTGTGC<br>R - GCCTCAGGCCTAAAACTCTACA | TD2 | 190 | 190 | 1 | 0 | 0 | 0 | all | Fluorescence | conserved hypothetical protein |
| EHBc-48 | $(CT)_{14}$ | F - CACGACGTTGTAAAACGACTCACCGAAGTTCATCGACAG<br>R - TTCCATTGATTGAGCATCCA | TD3 | 272 | 266-280 | 5 | 0.7576 | 0.5294 | 0.6889 | none | Fluorescence | Tetraspanin family protein |
| EHBc-49 | $(CAC)_5$ | F - CACGACGTTGTAAAACGACTGGATTGATGCTAGCTGTGG<br>R - GGCAACATATCCCCATCATT | TD3 | 196 | 187-196 | 2 | 0.5 | 0.5 | 0.368 | all | Fluorescence | transcription activator GLK1-like |
| EHBc-50 | $(TCT)_{11}$ | F - CACGACGTTGTAAAACGACCAATAATGGATGCAATCAGC<br>R - TTTGTTGCTGCACTCAGGAC | TD2 | 245 | 230-245 | 6 | 0.8079 | 0.8333 | 0.7507 | all | Fluorescence | rhodanese-like domain-containing family protein |
| EHBc-51 | $(TA)_8$ | F - CACGACGTTGTAAAACGACTGCAGACCTCATTTCCTTTG<br>R - ACAACAAATGCAAAACAAGC | TD2 | 215 | 210-245 | 5 | 0.369 | 0.2941 | 0.3439 | all | Fluorescence | conserved hypothetical protein |
| EHBc-52 | $(TTTC)_5$ | F - TTTTGTTCTTTCCGTTTCTGG<br>R - CAAGCGATGCAGGAGAAAA | 56 | 151 | 155 | 1 | 0 | 0 | 0 | all | Acrilamide | RNA-binding protein |
| EHBc-53 | $(CTT)_6$ | F - CTTTGTCGGATCCGCATTAC<br>R - CAACGTCAAAATCACCATGC | 62 | 184 | 177-180 | 2 | 0.4365 | 0.1667 | 0.3343 | all | Acrilamide | LMBR1 domain-containing protein 2 homolog A-like |
| EHBc-54 | $(TATT)_4$ | F - CACGACGTTGTAAAACGACACCGTTGTGCTTGTGGATA<br>R - TTGCTGCTGGATTTTCTTCA | TD2 | 210 | 210 | 1 | 0 | 0 | 0 | all | Fluorescence | Rhomboid-related intramembrane serine protease family protein, putative isoform 1 |
| EHBc-55 | $(GAT)_6$ | F - CACGACGTTGTAAAACGACGAAGAAACGGCCAAGAAGAA<br>R - CATCTCTTGAAAATGCCGAAA | TD2 | 181 | 157-181 | 3 | 0.1603 | 0.166 | 0.1494 | all | Fluorescence | conserved hypothetical protein |
| EHBc-56 | $(GAA)_5$ | F - CACGACGTTGTAAAACGACGGGTAGCCTTTGCCTAAACC<br>R - GGTGGTGATCCCTCTTCTT | TD2 | 173 | 173 | 1 | 0 | 0 | 0 | all | Fluorescence | conserved hypothetical protein |
| EHBc-57 | $(AG)_{16}$ | F - CACGACGTTGTAAAACGACACCTTCCCGGCTTTAATTG<br>R - GAGAGGGAGATGATGGGTGA | TD2 | 284 | 276-294 | 6 | 0.6532 | 0.6875 | 0.5661 | all | Fluorescence | conserved hypothetical protein |
| EHBc-58 | $(TAT)_6$ | F - CACGACGTTGTAAAACGACACAACCCTGTGGCTGTGACT<br>R - CATGTGCCCTTCTTGTGAGA | TD2 | 186 | 186-189 | 2 | 0.0556 | 0.0556 | 0.0526 | all | Fluorescence | homeobox-leucine zipper protein HAT14-like |
| EHBc-59 | $(TC)_{12}$ | F - CACGACGTTGTAAAACGACCTCGCGCTCTCTGAATCTTT | TD2 | 216 | 216-226 | 4 | 0.2587 | 0.0556 | 0.2412 | all | Fluorescence | nudix hydrolase 25-like |

| | | | | | | | | | | | | |
|---|---|---|---|---|---|---|---|---|---|---|---|---|
| | | R - GGCCTTATTGAAACCCAACA | | | | | | | | | | |
| EHBc-60 | $(AG)_{13}$ | F - CACGACGTTGTAAAACGACGAGAGGTAGATAGGCGGGAAA R - GGGTGATCCCAGAAACAGAA | TD1 | 267 | 267-280 | 4 | 0.5933 | 0.4 | 0.5362 | none | Fluorescence | conserved hypothetical protein |
| EHBc-61 | $(TTC)_5$ | F - CACGACGTTGTAAAACGACGGAGTTGAATCGGGTTTCTG<br>R - GGAAGCATGGGATTGAAAAA | TD1 | 231 | 231 | 1 | 0 | 0 | 0 | all | Fluorescence | Lorelei-like-gpi-anchored protein 1 |
| EHBc-62 | $(CT)_{10}$ | F - CACGACGTTGTAAAACGACTTCTTTGGTGGAATAGTTGGTTT<br>R - TGCAAGAGGGAGGAAGACTC | TD1 | 213 | 213-215 | 2 | 0.4978 | 0.5333 | 0.3739 | all | Fluorescence | pectin acetylesterase |
| EHBc-63 | $(GA)_6$ | F - CACGACGTTGTAAAACGACCTCAGTGGAGAGCGAAAATTG R - GCAATATCACATCCGTCATCC | TD1 | 253 | 253 | 1 | 0 | 0 | 0 | all | Fluorescence | PPPDE peptidase domain-containing protein |
| EHBc-64 | $(GAA)_6$ | F - TCTCCACTGCGGAGTCTTTT<br>R - ACCCCTAGGACCTGGTCAGT | 63 | 264 | 264-270 | 3 | 0.66 | 0.22 | 0.5675 | H. gui, H. rig, H. pau, H. bent, H. cam | Acrilamide | GATA domain class transcription factor |
| EHBc-65 | $(AC)_7(AT)_6$ | F - CCCCCTTCAGCCTTCAATA<br>R - TCCCCACTTCTCAAACAAGC | 60 | 241 | 241-249 | 3 | 0.53 | 0.33 | 0.4264 | H. gui, H. rig, H. nit | Acrilamide | cysteine-type peptidase, putative |
| EHBc-66 | $(ACA)_5G(CAG)_5$ | F - ACTTCTTGACCCACCCTCCT<br>R - GGCATCCTTATCCTCCTTCC | 63 | 250 | 250-259 | 3 | 0.63 | 0.89 | 0.5267 | H. gui, H. nit, H. pau, H. bent, H. cam | Capillary Electrophoresis | TCP domain class transcription factor |
| EHBc-67 | $(CGATTC)_7$ | F - TCGGACGGATGAAAAAGTTC<br>R - CAGGGTCATTTCCGTCATTT | 63 | 241 | 217-241 | 4 | 0.61 | 0.67 | 0.5122 | all | Acrilamide | BTB and TAZ domain protein 2 isoform 2 |
| EHBc-68 | $(ATT)_8$ | F - GTTGCGTTTTCTCGCAATTT<br>R - AGCCAAACAAGCACAGGAAT | 55 | 234 | 234-237 | 2 | 0.49 | 0 | 0.3624 | H. gui, H. bent, H. cam | Acrilamide | no hit |
| EHBc-69 | $(AT)_7(AG)_{11}G(GA)_8$ | F - AAAAAGGGAAAAATTTAGCAA<br>R - TCGCTTCAGTTCATCTGGTG | 55 | 177 | 177-201 | 6 | 0.76 | 0.5 | 0.7011 | all | Acrilamide | Hydroxyproline-rich glycoprotein family protein |
| EHBc-70 | $(CTT)_{10}$ | F - GGGGACGAGAAGCAAACATA<br>R - CACCCTTTTGAAAGCGAAAT | 60 | 162 | 150-171 | 4 | 0.63 | 0.61 | 0.5517 | all | Acrilamide | no hit |
| EHBc-71 | $(ATG)_6$ | F - CCACTGGCTCCAATCAAAAT<br>R - CAGCCTTGCTGTCATTCTCA | 63 | 264 | 332-359 | 4 | 0.53 | 0 | 0.4846 | all | Capillary Electrphosresis | exocyst complex component SEC3A isoform 1 |
| EHBc-72 | $(AACC)_4$ | F - CACGACGTTGTAAAACGACTTGCTCTCCCTCTACACCTACA<br>R - AAGCGTTTGTCGTCTCCAAG | TD2 | 266 | 266 | 1 | 0 | 0 | 0 | all | Fluorescence | conserved hypothetical protein |
| EHBc-73 | $(AG)_9$ | F - CACGACGTTGTAAAACGACGCATTTCCCTCAGAAAAAGTAGA<br>R - GCATGAGGACTAGGGCTGAG | TD2 | 228 | 224-238 | 3 | 0.5524 | 0.4444 | 0.4408 | all | Fluorescence | CASP-like protein |
| EHBc-74a | $(TCT)_6$ | F - CACGACGTTGTAAAACGACCACAGCTCTTGTATCATCACAGC<br>R - CCTCTGGCCTCTTTCTTCCT | TD2 | 170 | 167-170 | 2 | 0.3222 | 0.2778 | 0.2642 | all | Fluorescence | conserved hypothetical protein |
| EHBc-74b | $(AAG)_6$ | F - CACGACGTTGTAAAACGACTCCTCTTCCCTCCTTTCCTC<br>R - GGCGACAAGGAGAAACTCTG | TD2 | 246 | 243-247 | 4 | 0.4905 | 0.5 | 0.4367 | all | Fluorescence | |
| EHBc-75 | $(TCT)_7$ | F - CACGACGTTGTAAAACGACGGGGTGGCAGTTAATTCTGA<br>R - CCCACTTGAGACTCCACAAA | TD2 | 226 | 223-229 | 3 | 0.1603 | 0.1667 | 0.1494 | all | Fluorescence | conserved hypothetical protein |
| EHBc-76 | $(TTTG)_4$ | F - CGATCCTTGTCGAAGCAAA<br>R - ATCCATCGTCATCGTCATCA | 62 | 208 | 208 | 1 | 0 | 0 | 0 | all | Acrilamide | double-stranded RNA binding protein, putative |

| | | | | | | | | | | | | |
|---|---|---|---|---|---|---|---|---|---|---|---|---|
| EHBc-77 | (TA)$_{10}$ | F - AAGCTTGCCGTTGAAAAGAA<br>R - GATCATTACATTGCCCATCAA | 62 | 256 | 246-252 | 3 | 0.2556 | 0.1667 | 0.2335 | all | Acrilamide | ATP synthase subunit b', chloroplastic-like |
| EHBc-78 | (GAA)$_7$ | F - TCTTCACCTCGCCTTCTCTG<br>R - CCATTAAACCTGCATCAGCA | 62 | 227 | 221-233 | 3 | 0.1603 | 0.0556 | 0.1494 | H. gui, H. rig, H. pau, H. bent | Acrilamide | chaperone protein dnaJ 20, chloroplastic |
| EHBc-79 | (GCT)$_6$ | F - CACGACGTTGTAAAACGACCAAGGGGCACAGATTCACTT<br>R - TATCCGAGGGCTACAAGCAC | TD2 | 136 | 136-145 | 4 | 0.4905 | 0.2778 | 0.4067 | all | Fluorescence | DNA double-strand break repair rad50 ATPase, putative |
| EHBc-80 | (ATTTT)$_4$ | F - CACGACGTTGTAAAACGACGCTCATCCCTGGAGACTCTG<br>R - CCCCAAGAATTCAAAGTCCA | TD2 | 231 | 231 | 1 | 0 | 0 | 0 | H. pau | Fluorescence | xyloglucan endotransglycosylase/hydrolase |
| EHBc-81 | (TGA)$_5$ | F - TGTTCTGATCGATTCCTCCA<br>R - CACGCCCATTTCTGACTACA | 62 | 178 | 178-181 | 2 | 0.1571 | 0.0556 | 0.1411 | all | Acrilamide | r3h domain containing protein, putative |
| EHBc-82 | (ATG)$_6$ | F - CACGACGTTGTAAAACGACAGGGAGTGATGTTGCTCACC<br>R - TGAAGGAATCATGGGGAAGT | TD2 | 142 | 142 | 1 | 0 | 0 | 0 | all | Fluorescence | leucine-rich repeat containing protein, putative |
| EHBc-83a | (CTT)$_5$ | F - CACGACGTTGTAAAACGACCCCAAGAAAACAATCCAACC<br>R - GCTAAAGCTTGATGTGGAAGATG | TD2 | 184 | 184 | 1 | 0 | 0 | 0 | all | Fluorescence | predicted protein |
| EHBc-83b | (TTC)$_5$ | F - CACGACGTTGTAAAACGACACAAATTTGCACCCAGGTT<br>R - GTGGAAAAAGGGCAAAACAA | TD2 | 232 | 232 | 1 | 0 | 0 | 0 | all | Fluorescence | |
| EHBc-84a | (TCT)$_6$ | F - CACGACGTTGTAAAACGACGAAGTCTTCAGCAACATCATCTTC<br>R - CAAGCACCAAAACCACCTTT | TD2 | 217 | 217-223 | 2 | 0.0667 | 0.0667 | 0.0624 | H. gui, H. rig, H. nit, H. pau | Fluorescence | conserved hypothetical protein |
| EHBc-84b | (CTT)$_5$-(CAT)$_7$ | F - AAAGGTGGTTTTGGTGCTTG<br>R - GTCCATTCGCATGTGATGTC | 62 | 154 | 151-160 | 3 | 0.3794 | 0.4444 | 0.3368 | all | Acrilamide | |
| EHBc-85 | (CT)$_7$ | F - CACGACGTTGTAAAACGACAGTATCTCCCTCGCTCTG<br>R - GCGATGCCCTTCAACATTAT | TD2 | 157 | 400 | 1 | 0 | 0 | 0 | H. gui, H. rig, H. nit, H. bent, H. cam | Fluorescence | Monothiol glutaredoxin-4 |
| EHBc-86 | (TTA)$_6$ | F - CACGACGTTGTAAAACGACAAACAGCCTCTCTGCATGGT<br>R - AATTGCATTTGCCGTAAACC | TD2 | 259 | 253-259 | 2 | 0.1079 | 0 | 0.0994 | all | Fluorescence | ATP binding protein, putative |
| EHBc-87 | (AGC)$_6$ | F - CACGACGTTGTAAAACGACCCCTCTACATCTGGCTCTGC<br>R - TGTTGGGAAGTGACATGGAA | TD2 | 281 | 281-335 | 2 | 0.0556 | 0.0556 | 0.0526 | all | Fluorescence | protein TIME FOR COFFEE-like |
| EHBc-88 | (TTATAA)$_4$ | F - CACGACGTTGTAAAACGACTGGACCGCCTGTATAACTCC<br>R - GACGCTAATTTACACCGACCA | TD2 | 273 | 267-282 | 3 | 0.1095 | 0.1111 | 0.1037 | all | Fluorescence | zinc finger protein, putative |
| EHBc-89 | (TC)$_9$ | F CACGACGTTGTAAAACGACAGGCAACAAATCACCGAATC<br>R - TATTGGCTCCAGCACCTCTT | TD2 | 228 | 228-246 | 5 | 0.4698 | 0.5 | 0.434 | all | Fluorescence | probable ADP-ribosylation factor GTPase-activating protein AGD8-like |
| EHBc-90 | (TTC)$_7$ | F - CACGACGTTGTAAAACGACCAAGCCGATCAGATCCAAAT<br>R - TTCTGAAGCTCTTCGAGATCC | TD2 | 150 | 150-156 | 3 | 0.6578 | 0.7059 | 0.5629 | all | Fluorescence | conserved hypothetical protein |
| EHBc-91 | (TTG)$_5$ | F - CACGACGTTGTAAAACGACGATGTGGTTTGCCTCCTTGT<br>R - CACAAGTTTCGTCTGCCAGT | TD2 | 283 | 283 | 1 | 0 | 0 | 0 | all | Fluorescence | ABC transporter I family member 17 |
| EHBc-92 | (CT)$_9$ | F - CACGACGTTGTAAAACGACTCGTTCATCCACCTCATCAA<br>R - GGAAGTCAGCTTACCGACCA | TD3 | 278 | 276-294 | 5 | 0.4889 | 0.5 | 0.434 | all | Fluorescence | probable receptor-like protein kinase At5g15080-like |
| EHBc-93 | (GAAAAG)$_4$ | F - CACGACGTTGTAAAACGACTTTCCTTCCCATCTCTTTCG | TD2 | 192 | 186-192 | 2 | 0.5143 | 0.1111 | 0.375 | all | Fluorescence | ras-related protein RABA6b |

| | | | | | | | | | | | | |
|---|---|---|---|---|---|---|---|---|---|---|---|---|
| | | R - TTGATTTCCCAACAGCTGAA | | | | | | | | | | |
| EHBc-94 | (ATG)$_6$ | F - CACGACGTTGTAAAACGACGGTGGCGGCTAATATGGTTA<br>R - CCCAGTCCAAAGGAACATTG | TD2 | 262 | 256-262 | 2 | 0.2857 | 0.2222 | 0.2392 | all | Fluorescence | adenylate translocator |
| EHBc-95 | (AT)$_{11}$ | F - CACGACGTTGTAAAACGACGGCATTTCCCTCTCCTAAG<br>R - CGATACAAAGTCCCAATGGA | TD2 | 202 | 200-218 | 4 | 0.527 | 0.4444 | 0.473 | H. gui, H. nit, H. pau | Fluorescence | sigma factor sigB regulation protein rsbQ isoform |
| EHBc-96 | (TC)$_{14}$ | F - CACGACGTTGTAAAACGACCAAAGCAAGAGCAAGCCAGT<br>R - GGCCTCAAAATCTCAGTACCC | TD2 | 183 | 177-193 | 7 | 0.7905 | 0.8333 | 0.7364 | all | Fluorescence | conserved hypothetical protein |
| EHBc-97 | (CT)$_{17}$ | F - CACGACGTTGTAAAACGACACACAAGTGCCATCCTCTCC<br>R - CTTGGGAATCAAACGATGCT | TD2 | 188 | 171-195 | 7 | 0.7556 | 0.3333 | 0.6902 | H. gui, H. rig, H. nit, H. pau, H. bent | Fluorescence | conserved hypothetical protein |
| EHBc-98 | (TC)$_{19}$(TA)$_9$ | F - CACGACGTTGTAAAACGACCATTATCAAATCGCCGATCA<br>R - TCATTGCAAATCGAAAAGGA | TD3 | 278 | 274-298 | 5 | 0.7487 | 0.4706 | 0.6773 | all | Fluorescence | BTB/POZ and TAZ domain-containing protein 1-like |
| EHBc-99 | (TTC)$_5$ | F - CACGACGTTGTAAAACGACGAATCTAATATCCTACATTCTCATTCC<br>R - CGTTTATGTGCTTCCCTGGT | TD2 | 251 | 247-268 | 5 | 0.681 | 0.7222 | 0.6 | all | Fluorescence | microtubule-associated protein RP/EB family member 1-like isoform 2 |
| EHBc-100 | (ATT)$_5$ | F - CACGACGTTGTAAAACGACCGTTGGTGTCAATGTGAAGC<br>R - TTCAATGAGTGATCTACATGCAA | TD2 | 274 | 274-277 | 2 | 0.4278 | 0 | 0.329 | all | Fluorescence | conserved hypothetical protein |
| EHBc-101 | (TA)$_9$ | F - CACGACGTTGTAAAACGACGGCACTTTCACTGATGCAAA<br>R - TTTATTCACCCTTCAGCAGAT | TD2 | 175 | 169-175 | 3 | 0.5187 | 0.1765 | 0.4372 | all | Fluorescence | predicted protein |
| EHBc-102 | (TCG)$_5$ | F - GAGTCGGTGTCGGAGTCATT<br>R - GCCAAACATGCGATAAATCTT | 62 | 160 | 160 | 1 | 0 | 0 | 0 | all | Acrilamide | reticulon-like protein B2-like isoform 1 |
| EHBc-103 | (TC)$_8$TA(TC)$_8$ | F - CACGACGTTGTAAAACGACTTCTGCTTCAAAACCAAACAA<br>R - GAGAGAGGTGGCAAAGAGGA | TD1 | 212 | 180-210 | 6 | n/a | n/a | n/a | all | Fluorescence | F-box protein PP2-A13-like |
| EHBle-1 | (AAAT)$_4$ | F - CATATGCTCCCGGATAAAGG<br>R - CCTGCCCCTTTCCATTCTAT | 63 | 194 | 194 | 1 | 0 | 0 | 0 | all | Acrilamide | B3 domain-containing protein |
| EHBle-2a | (TTC)$_6$ | F - GGTGTTGAGGAATGCTGTTG<br>R - TGTGTTTTGTTGGCCTTTTG | 63 | 262 | 262-274 | 2 | 0.06 | 0.06 | 0.0526 | all | Acrilamide | no hit |
| EHBle-2b | (AAG)$_6$ | F - CACACAGCCCTACATGCAAT<br>R - GGGGTTTGTTGTGCTGTTCT | 63 | 223 | 223-235 | 3 | 0.59 | 0.56 | 0.5095 | all | Acrilamide | |
| EHBle-3 | (CT)$_{11}$ | F - CACGACGTTGTAAAACGACTCTATCTAGATCATCCACACAACC<br>R - TGGACGAGAACGAGAGAGAA | TD2 | 103 | 103-113 | 4 | 0.554 | 0.6111 | 0.4438 | H. gui, H. rig, H. nit, H. bent, H. cam | Fluorescence | actin depolymerizing factor 4 |
| EHBle-4 | (AT)$_7$ | F - GTGAGGTACGGCATGTAG<br>R - TGCAGATTAATTTTCCCATTGA | 62 | 251 | 198-252 | 4 | 0.3209 | 0 | 0.2973 | H. rig, H. bent | Acrilamide | probable pectinesterase/pectinesterase inhibitor |
| EHBle-5 | (GT)$_{11}$ | F - CACGACGTTGTAAAACGACTGGCCTTGTCCATTGAACT<br>R - GAACTTTGCAGCGGATTGTT | TD3 | 219 | 215-223 | 5 | 0.6881 | 0.7059 | 0.6228 | all | Fluorescence | Sulfiredoxin-1, putative |
| EHBa-1 | (TGC)$_7$ | F - AGGCCTGTATCAATGGCAAC<br>R - CTTGTACTGCTTTGCGGTGA | 62 | 257 | 338-356 | 5 | 0.7 | 0.18 | 0.6519 | all | Capillary electrophoresis | Jasmonate-zim-domain protein 12, putative isoform 1 |
| EHBa-2 | (AG)$_7$ | F - GCGACCTGTTCTCTCTCACC<br>R - TGGAAGCTAAGGATCCAAATG | 60 | 261 | 261-265 | 3 | 0.11 | 0.06 | 0.1037 | H. gui, H. rig, H. nit, H. bent, H. cam | Acrilamide | conserved hypothetical protein |

| | | | | | | | | | | | | |
|---|---|---|---|---|---|---|---|---|---|---|---|---|
| EHBa-3 | $(TAA)_6$ | F - CACGACGTTGTAAAACGACCGCTTGCTTAGCAGATGATG<br>R - TGGAGCCAATGAATAAATTTC | 63 | 206 | 221-279 | 5 | 0.2529 | 0.2 | 0.2378 | all | Acrilamide | predicted protein |
| EHBa-4 | $(ATCT)_5$ | F - CACGACGTTGTAAAACGACGCACGGTAAGGCTAGGGTTT<br>R - TGAGGAAGGAACGGAAGAGA | TD2 | 104 | 111-119 | 3 | 0.5317 | 0.4444 | 0.4091 | all | Fluorescence | wound-induced basic protein |
| EHBa-5 | $(GCT)_5$ | F - CACGACGTTGTAAAACGACGGCAAATCAAAGGAACAAGC<br>R - GAAACAACATTACACCACTCCAGA | TD2 | 205 | 205-208 | 2 | 0.0556 | 0.0556 | 0.0526 | all | Fluorescence | acyl-CoA-binding protein |
| EHBa-6 | $(TAT)_5$ | F - CACGACGTTGTAAAACGACTTTGTTGAAGACGTCTGTTTTGA<br>R - AAAGAAATACCACCAAGATGACA | TD2 | 267 | 267-288 | 2 | 0.5143 | 0.5556 | 0.375 | all | Fluorescence | stress-induced hydrophobic peptide |

Primer pairs number followed by letters a and b - belong to the same EST; H. gui - *Hevea guianensis* , H. rig - *Hevea rigidifolia* , H. nit - *Hevea nitida* , H. pau - *Hevea pauciflora* , H. bent - *Hevea benthamiana* , H. cam - *Hevea camargoana* ; n/a - not applicable

p - panel library EST, la - latex library EST, c - cold-stressed library EST, le - leaf library EST, a - contig
